# Transforming machine translation: a deep learning system reaches news translation quality comparable to human professionals

**DOI:** 10.1038/s41467-020-18073-9

**Published:** 2020-09-01

**Authors:** Martin Popel, Marketa Tomkova, Jakub Tomek, Łukasz Kaiser, Jakob Uszkoreit, Ondřej Bojar, Zdeněk Žabokrtský

**Affiliations:** 1 Faculty of Mathematics and Physics, Charles University, Prague, 121 16 Czech Republic; 2 Ludwig Cancer Research Oxford, University of Oxford, Oxford, OX1 2JD UK; 3 Department of Computer Science, University of Oxford, Oxford, OX1 3QD UK; 4 Google Brain, Mountain View, California, CA 94043 USA

**Keywords:** Computer science, Software, Communication

## Abstract

The quality of human translation was long thought to be unattainable for computer translation systems. In this study, we present a deep-learning system, CUBBITT, which challenges this view. In a context-aware blind evaluation by human judges, CUBBITT significantly outperformed professional-agency English-to-Czech news translation in preserving text meaning (translation adequacy). While human translation is still rated as more fluent, CUBBITT is shown to be substantially more fluent than previous state-of-the-art systems. Moreover, most participants of a Translation Turing test struggle to distinguish CUBBITT translations from human translations. This work approaches the quality of human translation and even surpasses it in adequacy in certain circumstances.This suggests that deep learning may have the potential to replace humans in applications where conservation of meaning is the primary aim.

## Abstract (web summary)

The quality of human language translation has been thought to be unattainable by computer translation systems. Here the authors present CUBBITT, a deep learning system that outperforms professional human translators in retaining text meaning in English-to-Czech news translation, and validate the system on English-French and English-Polish language pairs.

## Introduction

The idea of using computers for translation of natural languages is as old as computers themselves^1^. However, achieving major success remained elusive, in spite of the unwavering efforts of the machine translation (MT) research over the last 70 years. The main challenges faced by MT systems are correct resolution of the inherent ambiguity of language in the source text, and adequately expressing its intended meaning in the target language (translation adequacy) in a well-formed and fluent way (translation fluency). Among key complications is the rich morphology in the source and especially in the target language^2^. For these reasons, the level of human translation has been thought to be the upper bound of the achievable performance^3^. There are also other challenges in recent MT research such as gender bias^4^ or unsupervised MT^5^, which are mostly orthogonal to the present work.

Deep learning transformed multiple fields in the recent years, ranging from computer vision^6^ to artificial intelligence in games^7^. In line with these advances, the field of MT has shifted to the use of deep-learning neural-based methods^8–11^, which replaced previous approaches, such as rule-based systems^12^ or statistical phrase-based methods^13,14^. Relying on the vast amounts of training data and unprecedented computing power, neural MT (NMT) models can now afford to access the complete information available anywhere in the source sentence and automatically learn which piece is useful at which stage of producing the output text. This removal of past independence assumptions is the key reason behind the dramatic improvement of translation quality. As a result, neural translation even managed to considerably narrow the gap to human-translation quality on isolated sentences^15,16^.

In this work, we present a neural-based translation system CUBBITT (Charles University Block-Backtranslation-Improved Transformer Translation), which significantly outperformed professional translators on isolated sentences in a prestigious competition WMT 2018, namely the English–Czech News Translation Task^17^. We perform a new study with conditions that are more representative and far more challenging for MT, showing that CUBBITT conveys meaning of news articles significantly better than human translators even when the cross-sentence context is taken into account. In addition, we validate the methodological improvements using an automatic metric on English↔French and English↔Polish news articles. Finally, we provide insights into the principles underlying CUBBITT’s key technological advancement and how it improves the translation quality.

## Results

### Deep-learning framework transformer

**Fig. 1:**
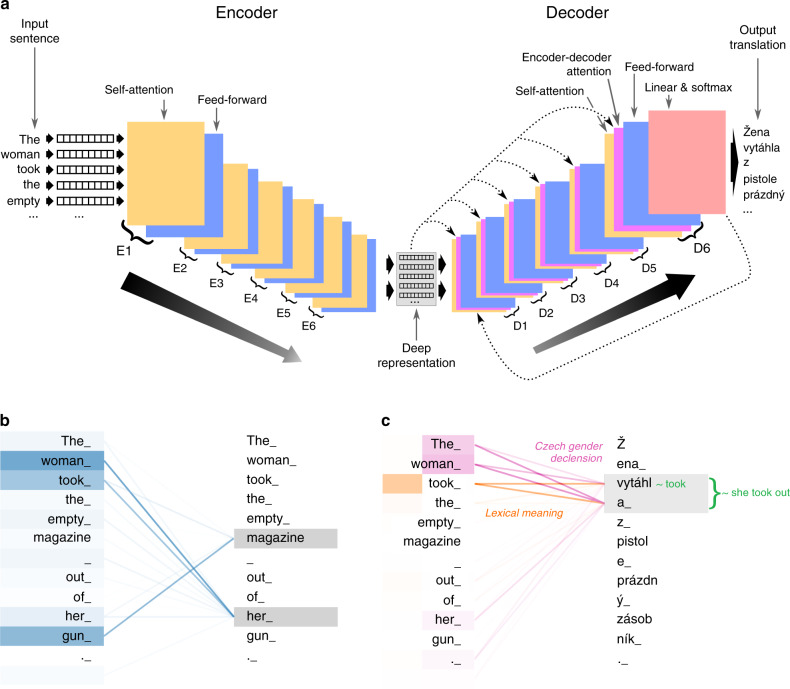
Model architecture of the CUBBITT machine translation system. **a** The input sentence is converted to a numerical representation and encoded into a deep representation by a six-layer encoder, which is subsequently decoded by a six-layer decoder into the translation in the target language. Layers of the encoder and decoder consist of self-attention and feed-forward layers and the decoder also contains an encoder-decoder attention layer, with an input of the deep representation created by the last layer of encoder. **b** Visualization of encoder self-attention between the first two layers (one attention head shown, focusing on “magazine” and “her”). The strong attention link between ‘magazine’ and ‘gun’ suggests why CUBBITT ultimately correctly translates “magazine” as “zásobník” (gun magazine), rather than “časopis” (e.g., news magazine). The attention link between ‘woman’ and ‘her’ illustrates how the system internally learns coreference. **c** Encoder-decoder attention on the second layer of the decoder. Two heads are shown in different colors, each focusing on a different translation aspect which is described in italic. We note that the attention weights were learned spontaneously by the network, not inputted a priori.

Our CUBBITT system (Methods 1) follows the basic Transformer encoder-decoder architecture introduced by Vaswani et al.^18^. The encoder represents subwords^19^ in the source-language sentence by a list of vectors, automatically extracting features describing relevant aspects and relationships in the sentence, creating a deep representation of the original sentence. Subsequently, the decoder converts the deep representation to a new sentence in the target language (Fig. 1a, Supplementary Fig. 1).

A critical feature of the encoder and decoder is self-attention, which allows identification and representation of relationships between sentence elements. While the encoder attention captures the relationship between the elements in the input sentence (Fig. 1b), the encoder-decoder attention learns the relationship between elements in the deep representation of the input sentence and elements in the translation (Fig. 1c). In particular, our system utilizes the so-called multi-head attention, where several independent attention functions are trained at once, allowing representation of multiple linguistic phenomena. These functions may facilitate, for example, the translation of ambiguous words or coreference resolution.

### Utilizing monolingual data via backtranslation

The success of NMT depends heavily on the quantity and quality of the training parallel sentences (i.e., pairs of sentences in the source and target language). Thanks to long-term efforts of researchers, large parallel corpora have been created for several language pairs, e.g., the Czech-English corpus CzEng^20^ or the multi-lingual corpus Opus^21^. Although millions of parallel sentences became freely available in this way, this is still not sufficient. However, the parallel data can be complemented by monolingual target-language data, which are usually available in much larger amounts than the parallel data. CUBBITT leverages the monolingual data using a technique termed backtranslation, where the monolingual target-language data are machine translated to the source language, and the resulting sentence pairs are used as additional (synthetic) parallel training data^19^. Since the target side in backtranslation are authentic sentences originally written in the target language, backtranslation can improve fluency (and sometimes even adequacy) of the final translations by naturally learning the language model of the target language.

**Fig. 2:**
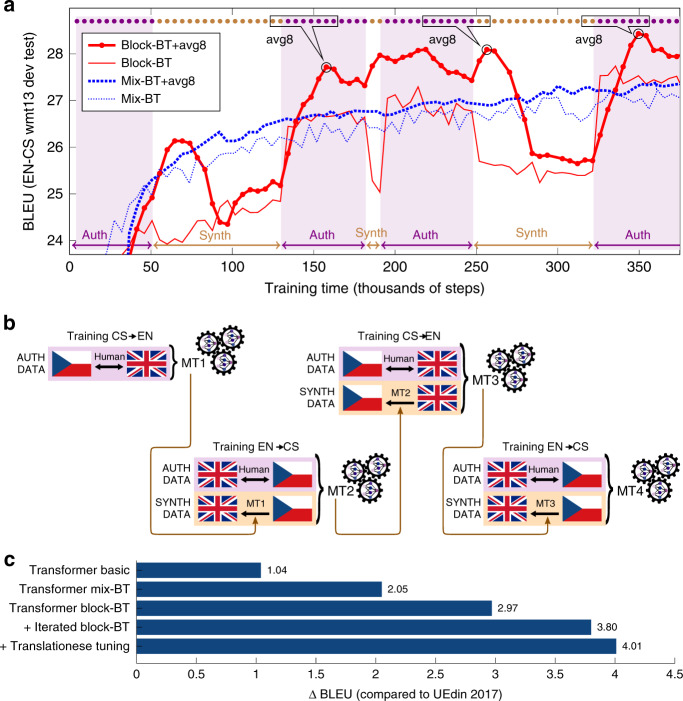
Backtranslation technique used in CUBBITT. **a** The effect of averaging eight last checkpoints with block-BT and mix-BT on the translation quality as measured by BLEU on the development set WMT13 newstest. The callouts (pointing to the initial and final peaks of the block-BT + avg8 curve) illustrate the 8 averaged checkpoints (synth-trained ones as brown circles, auth-trained ones as violet circles). **b** Diagram of iterated backtranslation: the system MT1 trained only on authentic parallel data is used to translate monolingual Czech data into English, which are used to train system MT2; this step can be iterated one or more times to obtain MT3, MT4, etc. The block-BT + avg8 model shown in **a** is the MT2 model in (B) and in Supplementary Fig. 2. **c** BLEU results on WMT17 test-set relative to the WMT17 winner UEdin2017. All five systems use checkpoint averaging.

CUBBITT is trained with backtranslation data in a novel block regime (block-BT), where the training data are presented to the neural network in blocks of authentic parallel data alternated with blocks of synthetic data. We compared our block regime to backtranslation using the traditional mixed regime (mix-BT), where all synthetic and authentic sentences are mixed together in random order, and evaluated the learning curves using BLEU, an automatic measure, which compares the similarity of an MT output to human reference translations (Methods 2–13). While training with mix-BT led to a gradually increasing learning curve, block-BT showed further improved performance in the authentic training phases, alternated with reduced performance in the synthetic ones (Fig. 2a, thin lines). In the authentic training phases, block-BT was better than mix-BT, suggesting that a model extracted at the authentic-data phase might perform better than mix-BT trained model.

CUBBITT combines block-BT with checkpoint averaging, where networks in the eight last checkpoints are merged together using arithmetic average, which is a very efficient approach to gain better stability, and by that improve the model performance^18^. Importantly, we observed that checkpoint averaging works in synergy with the block-BT. The BLEU improvement when using this combination is clearly higher than the sum of BLEU improvements by the two methods in separation (Fig. 2a). The best performance was gained when averaging authentic-trained model and synthetic-trained models in the ratio of 6:2; interestingly, the same ratio turned out to be optimal across several occasions in training. This also points out an advantage of block-BT combined with checkpoint averaging: the method automatically finds the optimal ratio of the two types of synthetic/authentic-trained models, as it evaluates all the ratios during training (Fig. 2a).

The final CUBBITT system was trained using iterated block-BT (Fig. 2b, Supplementary Fig. 2). This was accompanied by other steps, such as data filtering, translationese tuning, and simple regex postprocessing (Methods 11). Evaluating the individual components of CUBBITT automatically on a previously unseen test-set from WMT17 showed a significant improvement in BLEU over UEdin2017, the state-of-the-art system from 2017 (Fig. 2c).

### Evaluation: CUBBITT versus a professional agency translation

**Fig. 3:**
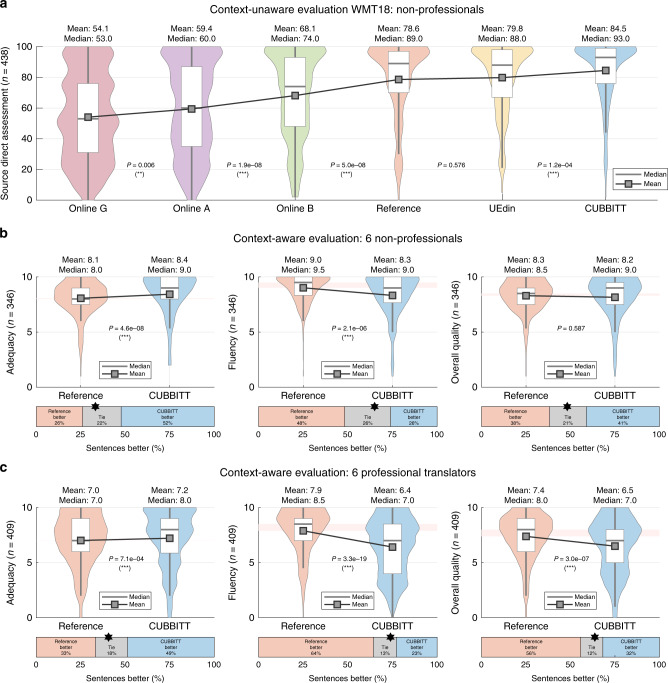
CUBBITT outperforms human translation in adequacy. **a** Results from context-unaware evaluation in WMT18, showing distributions of source-based direct assessment (SrcDA) of five MT systems and human reference translation, sorted by average score. CUBBITT was submitted under the name CUNI-Transformer. Online G, A, and B are three anonymized online MT systems. **b** Translations by CUBBITT and human reference were scored by six non-professionals in the terms of adequacy, fluency and overall quality in a context-aware evaluation. The evaluation was blind, i.e., no information was provided on whether the translations are human or machine translated. The scores (0–10) are shown as violin plots with boxplots (median + interquartile range), while the boxes below represent the percentage of sentences scored better in reference (orange), CUBBITT (blue), or the same (gray); the star symbol marks the ratio of orange vs. blue, ignoring gray. Sign test was used to evaluate difference between human and machine translation. **c** As in **a**, but evaluation by six professional translators. ***P < 0.001; **P < 0.01; *P < 0.05.

In 2018, CUBBITT won the English→Czech and Czech→English news translation task in WMT18^17^, surpassing not only its machine competitors, but it was also the only MT system, which significantly outperformed the reference human translation by a professional agency in WMT18 English→Czech news translation task (other language pairs were not evaluated in such a way to allow comparison with the human reference) (Fig. 3a). Since this result is highly surprising, we decided to investigate it in greater detail, evaluating potential confounding factors and focusing at how it can be explained and interpreted. We first confirmed that the results are not due to the original language of the reference sentences being English in half of the evaluated sentences and Czech in the other half of the test dataset (Supplementary Fig. 4; Methods 13), which was proposed to be a potential confounding factor by the WMT organizers^17^ and others^22,23^.

An important drawback in the WMT18 evaluation was the lack of cross-sentence context, as sentences were evaluated in random order and without document context. While the participating MT systems translated individual sentences independently, the human reference was created as a translation of the entire documents (news articles). The absence of cross-sentence context in the evaluation was recently shown to cause an overestimation of the quality of MT translations compared to human reference^22,23^. For example, evaluators will miss MT errors that would be evident only from the cross-sentence context, such as gender mismatch or incorrect translation of an ambiguous expression. On the other hand, independent evaluation of sentences translated considering cross-sentence context might unfairly penalize reference translations for moving pieces of information across sentences boundaries, as this will appear as an omission of meaning in one sentence and an addition in another.

We therefore conducted a new evaluation, using the same English→Czech test dataset of source documents, CUBBITT translations, and human reference translations, but presenting the evaluators with not only the evaluated sentences but also the document context (Methods 14–18; Supplementary Fig. 5). In order to gain further insight into the results, we asked the evaluators to assess the translations in terms of adequacy (the degree to which the meaning of the source sentence is preserved in the translation), fluency (how fluent the sentence sounds in the target language), as well as the overall quality of the translations. Inspired by a recent discussion of the translation proficiency of evaluators^22^, we recruited two groups of evaluators: six professional translators (native in the target language) and seven non-professionals (with excellent command of the source language and native in the target language). An additional exploratory group of three translation theoreticians was also recruited. In total, 15 out of the 16 evaluators passed a quality control check, giving 7824 sentence-level scores on 53 documents in total. See Methods 13–18 for further technical details of the study.

Focusing first at evaluations by non-professionals as in WMT18, but in our context-aware assessment, CUBBITT was evaluated to be significantly better than the human reference in adequacy (*P* = 4.6e-8, sign test) with 52% of sentences scored better and only 26% of sentences scored worse (Fig. 3b). On the other hand, the evaluators found human reference to be more fluent (*P* = 2.1e-6, sign test), evaluating CUBBITT better in 26% and worse in 48% (Fig. 3b). In the overall quality, CUBBITT nonsignificantly outperformed human reference (*P* = 0.6, sign test, 41% better than reference, 38% worse; Fig. 3b).

In the evaluation by professional translators, CUBBITT remained significantly better in adequacy than human reference (*P* = 7.1e-4, sign test, 49% better, 33% worse; Fig. 3c), albeit it scored worse in both fluency (*P* = 3.3e-19, sign test, 23% better, 64% worse) and overall quality (*P* = 3.0e-7, sign test, 32% better, 56% worse; Fig. 3c). Fitting a linear model of weighting adequacy and fluency in the overall quality suggests that professional translators value fluency more than non-professionals; this pattern was also observed in the exploratory group of translation theoreticians (Supplementary Fig. 6). Finally, when scores from all 15 evaluators were pooled together, the previous results were confirmed: CUBBITT outperformed the human reference in adequacy, whereas the reference was scored better in fluency and overall quality (Supplementary Fig. 7). Surprisingly, we observed a weak, but significant effect of sentence length, showing that CUBBITT’s performance is more favorable compared to human in longer sentences with regards to adequacy, fluency, and overall quality (Supplementary Fig. 8, including an example of a well-translated complex sentence).

We next decided to perform additional evaluation that would allow us to better understand where and why our machine translations are better or worse than the human translations. We asked three professional translators and three non-professionals to add annotations of types of errors in the two translations (Methods 19). In addition, the evaluators were asked to indicate whether the translation was wrong because of cross-sentence context.

**Fig. 4:**
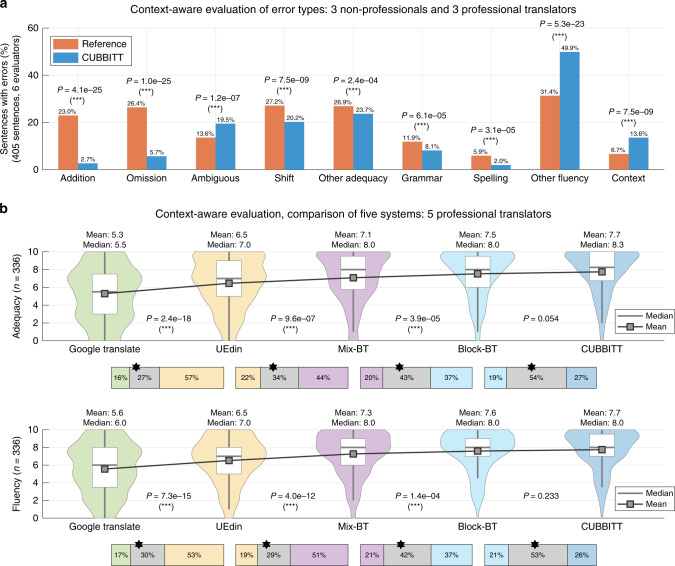
Types of errors made by CUBBITT versus human and a comparison of machine systems. **a** Percentages of sentences with various types of errors are shown for translations by human reference and CUBBITT. Errors in 405 sentences were evaluated by six evaluators (three professional translators and three non-professionals). Sign test was used to evaluate difference between human and machine translation. **b** Translations by five machine translation systems were scored by five professional translators in the terms of adequacy and fluency in a blind context-aware evaluation. The systems are sorted according to the mean performance, and the scores (0–10) for individual systems are shown as violin plots with boxplots (median + interquartile range). For each pair of neighboring systems, the box in between them represents the percentage of sentences scored as better in one, the other, or the same in both (gray). The star symbol marks the ratio when ties are ignored. Sign test was used to evaluate difference between the pairs of MT systems. ***P < 0.001; **P < 0.01; *P < 0.05.

**Fig. 5:**
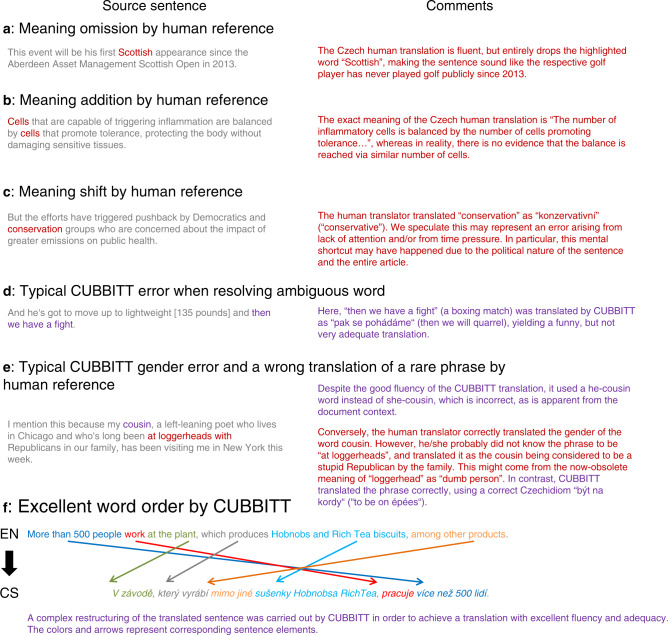
Examples of translation errors and properties of the reference human translation and CUBBITT. The Czech translations by the human reference and CUBBITT, as well as the values of the manual evaluation for the individual sentences, are shown in Supplementary Data 1.

CUBBITT made significantly fewer errors in addition of meaning, omission of meaning, shift of meaning, other adequacy errors, grammar, and spelling (Fig. 4a, example in Fig. 5a–c, Supplementary Data 1). On the other hand, reference performed better in error classes other fluency errors and ambiguous words (Fig. 4a, Supplementary Fig. 9, examples in Fig. 5d, e, Supplementary Data 1). As expected, CUBBITT made significantly more errors due to cross-sentence context (11.7% compared to 5.2% in reference, *P* = 1.2e-10, sign test, Fig. 4a), confirming the importance of context-aware evaluation of translation quality. Interestingly, when only sentences without context errors are taken into account, not only adequacy, but also the overall quality is significantly better in CUBBITT compared to reference in ratings by non-professionals (*P* = 0.001, sign test, 49% better, 29% worse; Supplementary Fig. 10), in line with the context-unaware evaluation in WMT18.

We observed that the type of document, e.g., business vs. sports articles, can also affect the quality of machine translation when compared to human translation (Methods 18). The number of evaluated documents (53) does not allow for any strong and significant conclusions at the level of whole documents, but the document-level evaluations nevertheless suggest that CUBBITT performs best in news articles about business and politics (Supplementary Fig. 11A-B). Conversely, it performed worst in entertainment/art (both in adequacy and fluency) and in news articles about sport (in fluency). Similar results can be observed also in sentence-level evaluations across document types (Supplementary Fig. 11C–D).

The fact that translation adequacy is the main strength of CUBBITT is surprising, as NMT was shown to improve primarily fluency over the previous approaches^24^. We were therefore interested in comparison of fluency of translations made by CUBBITT and previous state-of-the-art MT systems (Methods 20). We performed an evaluation of CUBBITT in a side-by-side direct comparison with Google Translate^15^ (an established benchmark for MT) and UEdin^25^ (the winning system in WMT2017 and a runner-up in WMT 2018). Moreover, we included a version of basic Transformer with one iteration of mix-BT, and another version of basic Transformer with block-BT (but without iterated block-BT), providing human rating of different approaches to backtranslation. The evaluators were asked to evaluate adequacy and fluency of the five presented translations (again in a blind setting and taking cross-sentence context into account).

In the context-aware evaluation of the five MT systems, CUBBITT significantly outperformed Google Translate and UEdin both in adequacy (mean increase by 2.4 and 1.2 points, respectively) and fluency (mean increase by 2.1 and 1.2 points, respectively) (Fig. 4b). The evaluation also shows that this increase of performance stems from inclusion of several components of CUBBITT: the Transformer model and basic (mix-BT) backtranslation, replacement of mix-BT with block-BT (adequacy: mean increase by 0.4, *P* = 3.9e-5; fluency: mean increase by 0.3, *P* = 1.4e-4, sign test), and to a lesser extent also other features in the final CUBBITT system, such as iterated backtranslation or data filtering (adequacy: mean increase by 0.2, *P* = 0.054; fluency: mean increase by 0.1, *P* = 0.233, sign test).

**Fig. 6:**
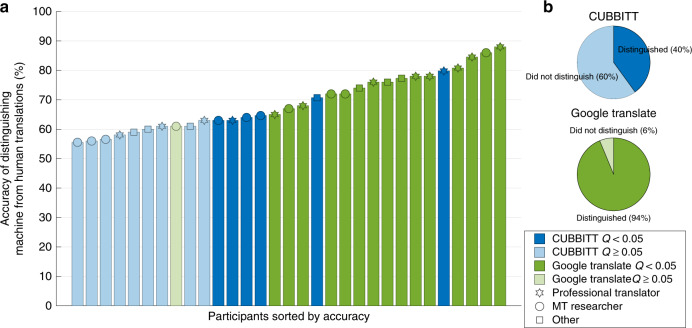
CUBBITT is difficult to distinguish from professional human translator in a Translation Turing test. **a** Accuracy of individual participants in distinguishing machine from human translations is shown in a bar graph. Fisher test was used to assess whether the participant significantly distinguished human and machine translations and Benjamini–Hochberg method was used to correct for multiple testing. Participants with a Q value below 0.05 were considered to have significantly distinguished between human and machine translations. **b** Percentage of participants, who significantly distinguished human and machine translations for CUBBITT (top, blue) and for Google Translate (bottom, green).

Finally, we were interested to see whether CUBBITT translations are distinguishable from human translations. We therefore conducted a sentence-level Translation Turing test, in which participants were asked to judge whether a translation of a sentence was performed by a machine or a human on 100 independent sentences (the source sentence and a single translation was shown; Methods 21). A group of 16 participants were given machine translations by Google Translate system mixed in a 1:1 ratio with reference translations. In this group, only one participant (with accuracy of 61%) failed to significantly distinguish between machine and human translations, while the other 15 participants recognized human translations in the test (with accuracy reaching as high as 88%; Fig. 6). In a group of different 15 participants, who were presented machine translations by CUBBITT mixed (again in the 1:1 ratio) with reference translations, nine participants did not reach the significance threshold of the test (with the lowest accuracy being 56%; Fig. 6). Interestingly, CUBBITT was not significantly distinguished from human translations by three professional translators, three MT researchers, and three other participants. One potential contributor to human-likeness of CUBBITT could be the fact that it is capable of restructuring translated sentences where the English structure would sound unnatural in Czech (see an example in Fig. 5f, Supplementary Data 1).

### Generality of block backtranslation

Block-BT with checkpoint averaging clearly improves English→Czech news translation quality. To demonstrate that the benefits of our approach are not limited to this language pair, we trained English→French, French→English, English→Polish, and Polish→English versions of CUBBITT (Methods 4, 5, 12) and evaluated them using BLEU as in Fig. 2a. The results are consistent with the behavior on the English→Czech language pair, showing a synergistic benefit of block-BT with checkpoint averaging (Fig. 2a, Supplementary Figs. 3, 14).

### How block backtranslation improves translation

Subsequently, we sought to investigate the synergy between block-BT and checkpoint averaging, trying to get an insight into the mechanism of how this improves translation on the English→Czech language pair. We first tested a simple hypothesis that the only benefit of block regime and checkpoint averaging is an automatic detection of the optimal ratio of authentic and synthetic data, given that in block-BT the averaging window explores various ratios of networks trained on authentic and synthetic data. Throughout our experiments, the optimal ratio of authentic and synthetic blocks was ca. 3:1, so we hypothesized that mixed-BT would benefit from authentic and synthetic data mixed in the same ratio. However, this hypothesis was not supported by additional explorations (Supplementary Fig. 15), which suggests that a more profound mechanism underlies the synergy.

We next hypothesized that training the system in the block regime compared to the mix regime might aid the network to better focus at the two types of blocks (authentic and synthetic), one at a time. This would allow the networks to more thoroughly learn the properties and benefits of the two blocks, leading to a better exploration of space of networks, ultimately yielding greater translation diversity during training. We measured translation diversity of a single sentence as the number of all unique translations produced by the MT system at hourly checkpoints during training. Comparing translation diversity between block-BT and mix-BT on the WMT13 newstest, we observed block-BT to have greater translation diversity in 78% sentences, smaller in 18% sentences, and equal in the remaining 4% sentences (Methods 22–23), supporting the hypothesis of greater translation diversity of block-BT compared to mix-BT.

**Fig. 7:**
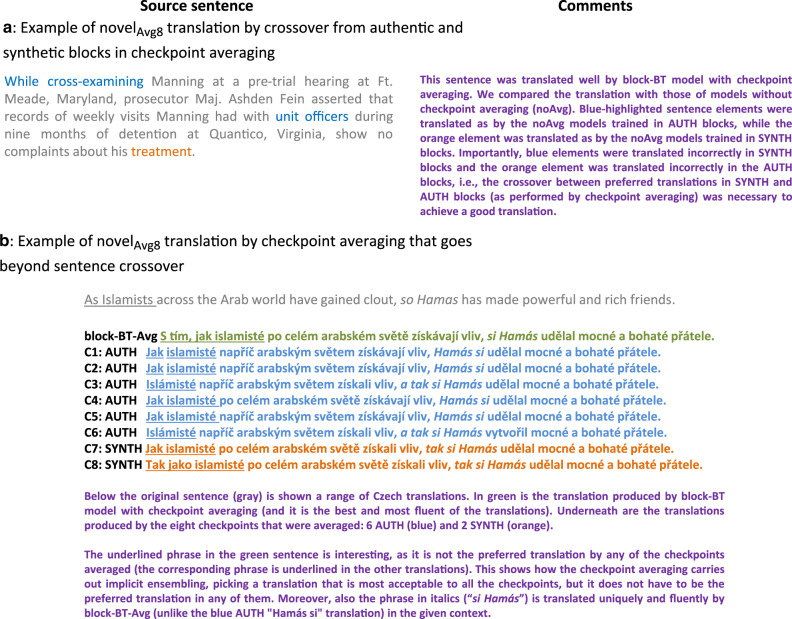
Examples of novel_Avg8_ sentences. **a** A case where the translation resulting from checkpoint averaging is a crossover of translations present in AUTH and SYNTH blocks. All the mentioned translations are shown in Supplementary Fig. 17. **b** A case where the translation resulting from checkpoint averaging contains a phrase that is not the preferred translation in any of the averaged checkpoints.

The increased diversity could be leveraged by checkpoint averaging by multiple means. In theory, this can be as simple as selecting the most frequent sentence translation among the eight averaged checkpoints. At the same time, checkpoint averaging can generate sentences that were not present as the preferred translation in any of the eight averaged checkpoints (termed novel_Avg8_ translation), potentially combining the checkpoints’ best translation properties. This may involve producing a combination of phrase translations seen in the averaged checkpoints (Fig. 7a, Supplementary Fig. 17), or creation of a sentence with phrases not seen in any of the averaged checkpoints (Fig. 7b). The fact that even phrase translations with low frequency in the eight averaged checkpoints can be chosen by checkpoint averaging stems from the way the confidence of the networks in their translations is taken into account (Supplementary Fig. 18).

Comparing the translations produced by models with and without averaging, we observed that averaging generated at least one translation never seen without averaging (termed novel_Avg∞_) in 60% sentences in block-BT and in 31.6% sentences in mix-BT (Methods 23). Moreover, averaging generated more novel_Avg∞_ translations in block-BT than mix-BT in 55% sentences, fewer in only 6%, and equal in 39%.

**Fig. 8:**
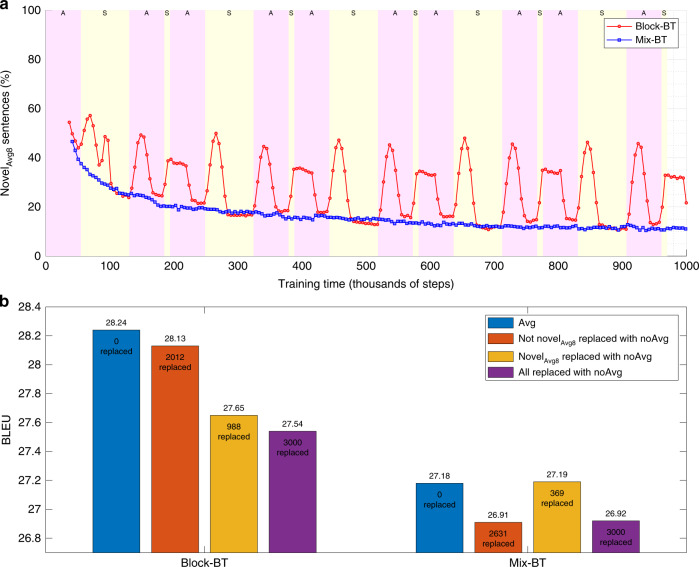
Synergy between block-BT and checkpoint averaging through novel_Avg8_ translations. **a** Percentage of WMT13 newstest sentences with novel_Avg8_ translation (not seen in the previous eight checkpoints without averaging) over time, shown separately for block-BT (red) and mix-BT (blue). The checkpoints trained in AUTH blocks are denoted by magenta background and letter A, while the SYNTH blocks are shown in yellow background and letter S. **b** Evaluation of translation quality by BLEU on WMT13 newstest set for four different versions of block-BT (left) and mix-BT (right), exploring the importance of novel_Avg8_ sentences created by checkpoint averaging. The general approach is to take the best system using checkpoint averaging (Avg), and substitute translations of novel_Avg8_ and not-novel_Avg8_ sentences with translations produced by the best system without checkpoint averaging (noAvg), observing the effect on BLEU. In blue is the BLEU achieved by the model with checkpoint averaging, while in purple is the BLEU achieved by the model without checkpoint averaging. In red is the BLEU of a system, which used checkpoint averaging, but where the translations that are not novel_Avg8_ were replaced by the translations produced by the system without checkpoint averaging. Conversely, yellow bars show BLEU of a system, which uses checkpoint averaging, but where the novel_Avg8_ translations were replaced by the version without checkpoint averaging.

We next sought to explore what is the mechanism of the greater translation diversity and more novel_Avg_ translations in block-BT compared to mix-BT. We therefore computed how translation diversity and novel_Avg8_ translations develop over time during training and what is their temporal relationship to blocks of authentic and synthetic data (Methods 24). In order to be able to track these features over time, we computed diversity and novel_Avg8_ using the last eight checkpoints (the width of the averaging window) for each checkpoint during training. While mix-BT gradually and smoothly decreased in both metrics over time, block-BT showed a striking difference between the alternating blocks of authentic and synthetic data (Fig. 8a, Supplementary Fig. 16). The novel_Avg8_ translations in block-BT were most frequent in checkpoints where the eight averaged checkpoints contained both the authentic- and synthetic-trained blocks (Fig. 8a). Interestingly, also the translation diversity of the octuples of checkpoints in block-BT (without averaging) was highest at the borders of the blocks (Supplementary Fig. 16). This suggests that it is the alternation of the blocks that increases the diversity of the translations and generation of novel translations by averaging in block-BT.

Finally, we tested whether the generation of novel translations by averaging contributes to the synergy between block regime and checkpoint averaging as measured by BLEU (Methods 25). We took the best model in block-BT with checkpoint averaging (block-BT-Avg; BLEU 28.24) and in block-BT without averaging (block-BT-NoAvg; BLEU 27.54). We next identified 988 sentences where the averaging in block-BT-Avg generated a novel_Avg8_ translation, unseen in the eight previous checkpoints without averaging. As we wanted to know what role do the novel_Avg8_ sentences play in the improved BLEU of block-BT-Avg compared to block-BT-NoAvg (Fig. 2a), we next computed BLEU of block-BT-Avg translations, where the translations of 988 novel_Avg8_ sentences were replaced with the block-BT-NoAvg translations. Such replacement led to decrease of BLEU almost to the level of block-BT-NoAvg (BLEU 27.65, Fig. 8b). Conversely, replacement of the 2012 not-novel_Avg8_ sentences resulted in only a small decrease (BLEU 28.13, Fig. 8b), supporting the importance of novel translations in the success of block-BT with checkpoint averaging. For a comparison, we repeated the same analysis with mix-BT and observed that replacement of novel_Avg8_ sentences in mix-BT showed a negligible effect on the improvement of mix-BT-Avg over mix-BT-NoAvg (Fig. 8b).

Altogether, our analysis shows that generation of novel sentences is an important mechanism of how checkpoint averaging combined with block-BT lead to synergistically improved performance. Specifically, averaging at the interface between authentic and synthetic blocks leads to the highest diversity and generation of novel translations, allowing combining the best features of the diverse translations in the two block types (examples in Fig. 7, Supplementary Fig. 17).

## Discussion

In this work, we have shown that the deep-learning framework CUBBITT outperforms a professional human-translation agency in adequacy of English→Czech news translations. In particular, this is achieved by making fewer errors in adding, omitting, or shifting meaning of the translated sentences. At the same time, CUBBITT considerably narrowed the gap in translation fluency to human, markedly outperforming previous state-of-the-art translation systems. The fact that the main advantage of CUBBITT is improved adequacy could be viewed as surprising, as it was thought that the main strength of NMT was increased fluency^24^. However, our results are in line with the study of Läubli et al.^23^, who observed the deficit of NMT to human to be smaller in adequacy than in fluency. The improvement in translation quality is corroborated by a Translation Turing test, where most participants failed to reliably discern CUBBITT translations from human.

Critically, our evaluation of translation quality was carried out in a fully context-aware evaluation setting. As discussed in this work and in other recent articles on this topic^22,23^, the previous standard approach of combining context-aware reference translation with context-free assessment gives an unfair advantage to machine translation. Consequently, this study is also an important contribution to MT evaluation practices and points out that the relevance of future evaluations in MT competitions such as WMT will be increased when cross-sentence context is included. In addition, our design where fluency and adequacy are assessed separately, and by professional translators and non-professionals, brings interesting insight into evaluator priorities. The professional translators were observed to be more sensitive to errors in fluency than non-professionals and to have a stronger preference for fluency when rating overall quality of a translation. Such difference in preference is an important factor in designing studies, which measure solely the overall translation quality. While in domains such as artistic writing, fluency is clearly of utmost importance, there are domains (e.g., factual news articles), where an improvement in preservation of meaning may be more important to a reader than a certain loss of fluency. Our robust context-aware evaluation with above-human performance in adequacy demonstrates that human translation is not necessarily an upper bound of translation quality, which was a long-standing dogma in the field.

Among key methodological advances of CUBBITT is the training regime termed block backtranslation, where blocks of authentic data alternate with blocks of synthetic data. Compared to traditional mixed backtranslation, where all the data are shuffled together, block regime offers markedly increased diversity of translations produced during training, suggesting a more explorative search for solutions to the translation problem. This increased diversity can be then greatly leveraged by the technique of checkpoint averaging, which is capable of finding consensus between networks trained on purely synthetic data and networks trained on authentic data, often combining the best of the two worlds. We speculate that such block-training regime of training may be beneficial also for other ways of data organization into blocks and may in theory be applicable beyond backtranslation, or even beyond the field of machine translation.

During reviews of this manuscript, the WMT19 competition took place^26^. The testing dataset was different, and evaluation methodology was innovated compared to WMT18, which is why the results are not directly comparable (e.g., the translation company was explicitly instructed to not add/remove information from the translated sentences, which was a major source of adequacy errors in this study (Fig. 4a)). Also based on discussions with our team’s members, the organizers of WMT19 implemented a context-aware evaluation. In this context-aware evaluation of English→Czech news task, CUBBITT was the winning MT system and reached overall quality score 95.3% of human translators (DA score 86.9 vs 91.2), which is similar to our study (94.8%, mean overall quality 7.4 vs 7.8, all annotators together). Given that WMT19 did not separate overall quality into adequacy and fluency, it is not possible to validate the potential super-human adequacy on their dataset.

Our study was performed on English→Czech news articles and we have also validated the methodological improvements of CUBBITT using automatic metric on English↔French and English↔Polish news articles. The generality of CUBBITT’s success with regards to other language pairs and domains remains to be evaluated. However, the recent results from WMT19 on English→German show that indeed also in other languages the human reference is not necessarily the upper bound of translation quality^26^.

The performance of machine translation is getting so close to human reference that the quality of the reference translation matters. Highly qualified human translators with infinite amount of time and resources will likely produce better translations than any MT system. However, many clients cannot afford the costs of such translators and instead use services of professional translation agencies, where the translators are under certain time pressure. Our results show that the quality of professional-agency translations is not unreachable by MT, at least in certain aspects, domains, and languages. Nevertheless, we suggest that in the future MT competitions and evaluations, it may be important to sample multiple human references (from multiple agencies and ideally also prices).

We stress out that CUBBITT is the result of years of open scientific collaboration and is a culmination of the transformation of the field. It started with the MT competitions that provided open data and ideas and continued through the open community of deep learning, which provided open-source code. The Transformer architecture significantly lowered the hardware requirements for training MT models (from months on multi-GPU clusters to days on a single machine^18^). More effective utilization of monolingual data via iterated block backtranslation with checkpoint averaging presented in this study allows generating large amount of high-quality synthetic parallel data to complement existing parallel datasets at little cost. Together, these techniques allow CUBBITT to be trained by the broad community and to considerably extend the reach of MT.

## Methods

### 1 CUBBITT model

Our CUBBITT translation system follows the Transformer architecture (Fig. 1, Supplementary Fig. 1) introduced in Vaswani et al.^18^. Transformer has an encoder-decoder structure where the encoder maps an input sequence of tokens (words or subword units) to a sequence of continuous deep representations *z*. Given *z*, the decoder generates an output sequence of tokens one element at a time. The decoder is autoregressive, i.e., consuming the previously generated symbols as additional input when generating the next token.

The encoder is composed of a stack of identical layers, with each layer having two sublayers. The first is a multi-head self-attention mechanism, and the second is a simple, position-wise fully connected feed-forward network. We employ a residual connection around each of the two sublayers, followed by layer normalization. The decoder is also composed of a stack of identical layers. In addition to the two sublayers from the encoder, the decoder inserts a third sublayer, which performs multi-head attention over the output of the encoder stack. Similar to the encoder, we employ residual connections around each of the sublayers, followed by layer normalization.

The self-attention layer in the encoder and decoder performs multi-head dot-product attention, each head mapping matrices of queries (*Q*), keys (*K*), and values (*V*) to an output vector, which is a weighted sum of the values *V*:

1 $${\mathrm{Attention}}\left( {Q,K,V} \right) = {\mathrm{softmax}}\left( {\frac{{QK^T}}{{\sqrt {d_k} }}} \right)V,$$

where *Q* ∈ ${\Bbb R}^{n \times d_k}$, *K* ∈ ${\Bbb R}^{n \times d_k}$, *V* ∈ ${\Bbb R}^{n \times d_v}$, *n* is the sentence length, *d*_*v*_ is the dimension of values, and *d*_*k*_ is the dimension of the queries and keys. Attention weights are computed as a compatibility of the corresponding key and query and represent the relationship between deep representations of subwords in the input sentence (for encoder self-attention), output sentence (for decoder self-attention), or between the input and output sentence (for encoder-decoder attention). In encoder and decoder self-attention, all queries, keys and values come from the output of the previous layer, whereas is the encoder-decoder attention, keys and values come from the encoder’s topmost layer and queries come from the decoder’s previous layer. In the decoder, we modify the self-attention to prevent it from attending to following positions (i.e., rightward from the current position) by adding a mask, because the following positions will not be known in inference time.

### 2 English–Czech training data

Our training data are constrained to the data allowed in the WMT 2018 News translation shared task^17^ (www.statmt.org/wmt18/translation-task.html). Parallel (authentic) data are: CzEng 1.7, Europarl v7, News Commentary v11 and CommonCrawl. Monolingual data for backtranslation are: English (EN) and Czech (CS) NewsCrawl articles. Data sizes (after filtering, see below) are reported in Supplementary Table 1.

While all our monolingual data are news articles, only less than 1% of our parallel data are news (summing News Commentary v12 and the news portion of CzEng 1.7). The biggest sources of our parallel data are: movie subtitles (63% of sentences), EU legislation (16% of sentences), and Fiction (9% of sentences)^27^. Unfortunately, no finer-grained metadata specifying the exact training-data domains (such as politics, business, and sport) are available.

We filtered out ca. 3% of sentences in the monolingual data by restricting the length to 500 characters and in case of Czech NewsCrawl also by keeping only sentences containing at least one accented character (using a regular expression m/[ěščřžýáíéúůd’t’ň]/i). This simple heuristic is surprisingly effective for Czech; it filters out not only sentences in other languages than Czech, but also various non-linguistic content, such as lists of football or stock-market results.

We divided the Czech NewsCrawl (synthetic data) into two parts: years 2007–2016 (58,231 k sentences) and year 2017 (7152 k sentences). When training block-BT, we simply concatenated four blocks of training data: authentic, synthetic 2007–2016, authentic and synthetic 2017. The sentences within these four blocks were randomly shuffled; we only do not shuffle across the data blocks. When training mix-BT, we used exactly the same training sentences, but we shuffled them fully. This means we upsampled the authentic training data two times. The actual ratio of authentic and synthetic data (as measured by the number of subword tokens) in the mix-BT training data was approximately 1.2:1.

### 3 English–Czech development and test data

WMT shared task on news translation provides a new test-set (with ~3000 sentences) each year collected from recent news articles (WMT = Workshop on statistical Machine Translation. In 2016, WMT was renamed to Conference on Machine Translation, but keeping the legacy abbreviation WMT. For more information see the WMT 2018 website http://www.statmt.org/wmt18.). The reference translations are created by professional translation agencies. All of the translations are done directly, and not via an intermediate language. Test sets from previous years are allowed to be used as development data in WMT shared tasks.

We used WMT13 (short name for WMT newstest2013) as the primary development set in our experiments (e.g., Figure 2a). We used WMT17 as a test-set for measuring BLEU scores in Fig. 2c. We used WMT18 (more precisely, its subset WMT18-orig-en, see below) as our final manual-evaluation test-set. Data sizes are reported in Supplementary Table 2.

In WMT test sets since 2014, half of the sentences for a language pair X-EN originate from English news servers (e.g., bbc.com) and the other half from X-language news servers. All WMT test sets include the server name for each document in metadata, so we were able to split our dev and test sets into two parts: originally Czech (orig-cs, for Czech-domain articles, i.e., documents with docid containing “.cz”) and originally English (orig-en, for non-Czech-domain articles. The WMT13-orig-en part of our WMT13 development set contains not only originally English articles, but also articles written originally in French, Spanish, German and Russian. However, the Czech reference translations were translated from English. In WMT18-orig-en, all the articles were originally written in English.).

According to Bojar et al.^17^, the Czech references in WMT18 were translated from English “by the professional level of service of Translated.net, preserving 1–1 segment translation and aiming for literal translation where possible. Each language combination included two different translators: the first translator took care of the translation, the second translator was asked to evaluate a representative part of the work to give a score to the first translator. All translators translate towards their mother tongue only and need to provide a proof or their education or professional experience, or to take a test; they are continuously evaluated to understand how they perform on the long term. The domain knowledge of the translators is ensured by matching translators and the documents using T-Rank, http://www.translated.net/en/T-Rank.”

Toral et al.^22^ furthermore warned about post-edited MT used as human references. However, Translated.net confirmed that MT was completely deactivated during the process of creating WMT18 reference translations (personal communication).

### 4 English–French data

The English–French parallel training data were downloaded from WMT2014 (http://statmt.org/wmt14/translation-task.html). The monolingual data were downloaded from WMT 2018 (making sure there is no overlap with the development and test data). We filtered the data for being English/French using the langid toolkit (http://pypi.org/project/langid/). Data sizes after filtering are reported in Supplementary Table 3. When training English–French block-BT, we concatenated the French NewsCrawl2008–2014 (synthetic data) and authentic data, with no upsampling. When training French–English block-BT, we split the English NewsCrawl into three parts: 2011–2013, 2014–2015, and 2016–2017 and interleaved with three copies of the authentic training data, i.e., upsampling the authentic data three times. We always trained mix-BT on a fully shuffled version of the data used for the respective block-BT training.

Development and test data are reported in Supplementary Table 4.

### 5 English–Polish data

The English–Polish training and development data were downloaded from WMT2020 (http://statmt.org/wmt20/translation-task.html). We filtered the data for being English/Polish using the FastText toolkit (http://pypi.org/project/fasttext/). Data sizes after filtering are reported in Supplementary Table 5. When training English–Polish block-BT, we upsampled the authentic data two times and concatenated with the Polish NewsCrawl2008–2019 (synthetic data) upsampled six times. When training Polish–English block-BT, we upsampled the authentic data two times and concatenated with English NewsCrawl2018 (synthetic data, with no upsampling). We always trained mix-BT on a fully shuffled version of the data used for the respective block-BT training.

Development and test data are reported in Supplementary Table 6.

### 6 CUBBITT training: BLEU score

BLEU^28^ is a popular automatic measure for MT evaluation and we use it for hyperparameter tuning. Similarly to most other automatic MT measures, BLEU estimates the similarity between the system translation and the reference translation. BLEU is based on n-gram (unigrams up to 4-grams) precision of the system translation relative to the reference translation and a brevity penalty to penalize too short translations. We report BLEU scaled to 0–100 as is usual in most papers (although BLEU was originally defined as 0–1 by Papineni et al.^28^); the higher BLEU value, the better translation. We use the SacreBLEU implementation^29^ with signature BLEU+case.mixed+lang.en-cs+numrefs.1+smooth.exp+tok.13a.

### 7 CUBBITT training: hyperparameters

We use the Transformer “big” model from the Tensor2Tensor framework v1.6.0^18^. We followed the training setup and tips of Popel and Bojar^30^ and Popel et al.^31^, training our models with the Adafactor optimizer^32^ instead of the default Adam optimizer. We use the following hyperparameters: learning_rate_schedule = rsqrt_decay, batch_size = 2900, learning_rate_warmup_steps = 8000, max_length = 150, layer_prepostprocess_dropout = 0, optimizer = Adafactor. For decoding, we use alpha = 1.0, beam_size = 4.

### 8 CUBBITT training: checkpoint averaging

A popular way of improving the translation quality in NMT is ensembling, where several independent models are trained and during inference (decoding, translation) each target token (word) is chosen according to an averaged probability distribution (using argmax in the case of greedy decoding) and used for further decisions in the autoregressive decoder of each model.

However, ensembling is expensive both in training and inference time. The training time can be decreased by using checkpoint ensembles^33^, where N last checkpoints of a single training run are used instead of N independently trained models. Checkpoint ensembles are usually worse than independent ensembles^33^, but allow to use more models in the ensemble thanks to shorter training time. The inference time can be decreased by using checkpoint averaging, where the weights (learned parameters of the network) in the N last checkpoints are element-wise averaged, creating a single averaged model.

Checkpoint averaging has been first used in NMT by Junczys-Dowmunt et al.^34^, who report that averaging four checkpoints is “not much worse than the actual ensemble” of the same four checkpoints and it is better than ensembles of two checkpoints. Averaging ten checkpoints “even slightly outperforms the real four-model ensemble”.

Checkpoint averaging has been popular in recent NMT systems because it has almost no additional cost (averaging takes only several minutes), the results of averaged models have lower variance in BLEU and are usually at least slightly better than models without averaging^30^.

In our experiments, we store checkpoints each hour and average the last 8 checkpoints.

### 9 CUBBITT training: Iterated backtranslation

For our initial experiments with backtranslation, we reused an existing CS → EN system UEdin (Nematus software trained by a team from the University of Edinburgh and submitted to WMT 2016^35^). This system itself was trained using backtranslation. We decided to iterate the backtranslation process further by using our EN → CS Transformer to translate English monolingual data and use that for training a higher quality CS → EN Transformer, which was in turn used for translating Czech monolingual data and training our final EN → CS Transformer system called CUBBITT. Supplementary Fig. 2 illustrates this process and provides details about the training data and backtranslation variants (mix-BT in MT1 and block-BT in MT2–4) used.

Each training we did (MT3–5 in Supplementary Fig. 2) took ca. eight days on a single machine with eight GTX 1080 Ti GPUs. Translating the monolingual data with UEdin2016 (MT0) took ca. two weeks and with our Transformer models (MT1–3) it took ca. 5 days.

### 10 CUBBITT training: translationese tuning

It has been observed that text translated from language X into Y has different properties (such as lexical choice or syntactic structure) compared to text originally written in language Y^36^. Term translationese is used in translation studies (translatology) for this phenomenon (and sometimes also for the translated language itself).

We noticed that when training on synthetic data, the model performs much better on the WMT13-orig-cs dev set than on the WMT13-orig-en dev set. When trained on authentic data, it is the other way round. Intuitively, this makes sense: The target side of our synthetic data are original Czech sentences from Czech newspapers, similarly to the WMT13-orig-cs dataset. In our authentic parallel data, over 90% of sentences were originally written in English about non-Czech topics and translated into Czech (by human translators), similarly to the WMT13-orig-en dataset. There are two closely related phenomena: a question of domain (topics) in the training data and a question of so-called translationese effect, i.e., which side of the parallel training data (and test data) is the original and which is the translation.

Based on these observations, we prepared an orig-cs-tuned model and an orig-en-tuned model. Both models were trained in the same way; they differ only in the number of training steps. For the orig-cs-tuned model, we selected a checkpoint with the best performance on WMT13-orig-cs (Czech-origin portion of WMT newstest2013), which was at 774k steps. Similarly, for the orig-en-tuned model, we selected the checkpoint with the best performance on WMT13-orig-en, which was at 788k steps. Note that both the models were trained jointly in one experiment, just selecting checkpoints at two different moments. The WMT18-orig-en test-set was translated using the orig-en-tuned model and the WMT18-orig-cs part was translated using the orig-cs-tuned model.

### 11 CUBBITT training: regex postediting

We applied two simple post-processings to the translations, using regular expressions. First, we converted quotation symbols in the translations to the correct-Czech lower and upper quotes („ and “) using two regexes: s/(ˆ|[({[])(“|,,|”|“)/$1„/g and s/(“|”)($|[,.?!:;)}\]])/“$2/g. Second, we deleted phrases repeated more than twice (immediately following each other); we kept just the first occurrence. We considered phrases of one up to four words. This postprocessing affected less than 1% sentences in the dev set.

### 12 CUBBITT training: English–French and English–Polish

We trained English→French, French→English, English→Polish and Polish→English versions of CUBBITT, following the abovementioned English–Czech setup, but using the training data described in Supplementary Tables 3 and 5 and the training diagram in Supplementary Fig. 3. All systems (including M1 and M2) were trained with Tensor2Tensor Transformer (no Nematus was involved). Iterated backtranslation was tried only for French→English. No translationese tuning was used (because we report just the BLEU training curve, but no experiments where the final checkpoint selection is needed). No regex post-diting was used.

### 13 Reanalysis of context-unaware evaluation in WMT18

We first reanalyzed results from the context-unaware evaluation of WMT 2018 English–Czech News Translation Task, provided to us by the WMT organizers (http://statmt.org/wmt18/results.html). The data shown in Fig. 3a were processed in the same way as by the WMT organizers: scores with BAD and REF types were first removed, a grouped score was computed as an average score for every triple language pair (“Pair”), MT system (“SystemID”), and sentence (“SegmentID”) was computed, and the systems were sorted by their average score. In Fig. 3a, we show distribution of the grouped scores for each of the MT systems, using paired two-tailed sign test to compare significance of differences of the subsequent systems.

We next assessed whether the results could be confounded by the original language of the source. Specifically, one half of the test-set sentences in WMT18 were originally English sentences translated to Czech by a professional agency, while the other half were English translations of originally Czech sentences. However, both types of sentences were used together for evaluation of both translation directions in the competition. Since the direction of translation could affect the evaluation, we first re-evaluated the MT systems in WMT18 by splitting the test-set according to the original language in which the source sentences were written.

Although the absolute values of source direct assessment were lower for all systems and reference translation in originally English source sentences compared to originally Czech sentences, CUBBITT significantly outperformed the human reference and other MT systems in both test sets (Supplementary Fig. 4). We checked that this was true also when comparing z-score normalized scores and using unpaired one-tail Mann–Whitney U test, as by the WMT organizers.

Any further evaluation in our study was performed only on documents with the source side as the original text, i.e., with originally English sentences in the English→Czech evaluations.

### 14 Context-aware evaluation: methodology

Three groups of paid evaluators were recruited: six professional translators, three translation theoreticians, and seven other evaluators (non-professionals). All 16 evaluators were native Czech speakers with excellent knowledge of the English language. The professional translators were required to have at least 8 years of professional translation experience and they were contacted via The Union of Interpreters and Translators (http://www.jtpunion.org/). The translation theoreticians were from The Institute of Translation Studies, Charles University’s Faculty of Arts (https://utrl.ff.cuni.cz/). Guidelines presented to the evaluators are given in Supplementary Methods 1.1.

For each source sentence, evaluators compared two translations: Translation T1 (the left column of the annotation interface) vs Translation T2 (the right column of the annotation interface). Within one document (news article), Translation T1 was always a reference and Translation T2 was always CUBBITT, or vice versa (i.e., each column within one document being purely reference translation or purely CUBBITT). However, evaluators did not know which system is which, nor that one of them is a human translation and the other one is a translation by MT system. The order of reference and CUBBITT was random in each document. Each evaluator encountered reference being Translation T1 in approximately one half of the documents.

Evaluators scored 10 consecutive sentences (or the entire document if shorter than 10 sentences) from a random section of the document (the same section was used in both T1 and T2 and by all evaluators scoring this document), but they had access to the source side of the entire document (Supplementary Fig. 5).

Every document was scored by at least two evaluators (2.55 ± 0.64 evaluators on average). The documents were assigned to evaluators in such a way that every evaluator scored nine different nonspam documents and most pairs of evaluators had at least one document in common. This maximized the diversity of annotator pairs in the computation of interannotator agreement. In total, 135 (53 unique) documents and 1304 (512 unique) sentences were evaluated by the 15 evaluators who passed quality control (see below).

### 15 Context-aware evaluation: quality control

The quality control check of evaluators was performed using a spam document, similarly as in Läubli et al.^23^ and Kittur et al.^37^. In MT translations of the spam document, the middle words (i.e., except the first and last words in the sentence) were randomly shuffled in each of the middle six sentences of the document (i.e., the first and last two sentences were kept intact). We ascertained that the resulting spam translations made no sense.

The criterion for evaluators to pass the quality control was to score at least 90% of reference sentences better than all spam sentences (in each category: adequacy, fluency, overall). One non-professional evaluator did not pass the quality control, giving three spam sentences a higher score than 10% of the reference sentences. We excluded the evaluator from the analysis of the results (but the key results reported in this study would hold even when including the evaluator).

### 16 context-aware evaluation: interannotator agreement

We used two methods to compute interannotator agreement (IAA) on the paired scores (CUBBITT—reference difference) in adequacy, fluency, and overall quality for the 15 evaluators. First, for every evaluator, we computed Pearson and Spearman correlation of his/her scores on individual sentences with a consensus of scores from all other evaluators. This consensus was computed for every sentence as the mean of evaluations by other evaluators who scored this sentence. This correlation was significant after Benjamini–Hochberg correction for multiple testing for all evaluators in adequacy and fluency and overall quality. The median and interquartile range of the Spearman *r* of the 15 evaluators were 0.42 (0.33–0.49) for adequacy, 0.49 (0.35–0.55) for fluency, and 0.49 (0.43–0.54) for overall quality. The median and interquartile range of the Pearson *r* of the 15 evaluators were 0.42 (0.32–0.49) for adequacy, 0.47 (0.39–0.55) for fluency, and 0.46 (0.40–0.50) for overall quality.

Second, we computed Kappa in the same way as in WMT 2012–2016^38^, separately for adequacy, fluency, and overall quality (Supplementary Table 7).

### 17 Context-aware evaluation: statistical analysis

First, we computed the average score for every sentence from all evaluators who scored the sentence within the group (non-professionals, professionals, translation theoreticians for Fig. 3 and Supplementary Fig. 7B) or within the entire cohort (for Supplementary Fig. 7A). The difference between human reference and CUBBITT translations was assessed using paired two-tailed sign test (Matlab function sign test) and *P* values below 0.05 were considered statistically significant.

In the analysis of relative contribution of adequacy and fluency in the overall score (Supplementary Fig. 6), we fitted a linear model through scores in all sentences, separately for human reference translations and CUBBITT translations for every evaluator, using matlab function fitlm(tableScores,‘overall~adequacy+fluency’,‘RobustOpts’,‘on’, ‘Intercept’, false).

### 18 Context-aware evaluation: analysis of document types

For analysis of document types (Supplementary Fig. 11), we grouped the 53 documents (news articles) into seven classes: business (including economics), crime, entertainment (including art, film, one article about architecture), politics, scitech (science and technology), sport, and world. Then we compared the relative difference of human reference minus CUBBITT translation scores on the document-level scores and sentence-level scores and used sign test to assess the difference between the two translations.

### 19 Evaluation of error types in context-aware evaluation

Three non-professionals and three professional translator evaluators performed a follow-up evaluation of error types, after they finished the basic context-aware evaluation. Nine columns were added into the annotation sheets next to their evaluations of quality (adequacy, fluency, and overall quality) of each of the two translations. The evaluators were asked to classify all translation errors into one of eight error types and to identify sentences with an error due to cross-sentence context (see guidelines). In total, 54 (42 unique) documents and 523 (405 unique) sentences were evaluated by the six evaluators. Guidelines presented to the evaluators are given in Supplementary Methods 1.2.

Similarly to Section 5.4, we compute IAA Kappa scores for each error type, based on the CUBBITT—Reference difference (Supplementary Table 8).

When carrying out statistical analysis, we first grouped the scores of sentences with multiple evaluations by computing the average number of errors per sentence and error type from the scores of all evaluators who scored this sentence. Next, we compared the percentage of sentences with at least one error (Fig. 4a) and the number of errors per sentence (Supplementary Fig. 9), using sign test to compare the difference between human reference and CUBITT translations.

### 20 Evaluation of five MT systems

Five professional-translator evaluators performed this follow-up evaluation after they finished the previous evaluations. For each source sentence, the evaluators compared five translations by five MT systems: Google Translate from 2018, UEdin from 2018, Transformer trained with one iteration of mix-BT (as MT2 in Supplementary Fig. 2, but with mix-BT instead of block-BT), Transformer trained with one iteration of block-BT (MT2 in Supplementary Fig. 2), and the final CUBBITT system. Within one document, the order of the five systems was fixed, but it was randomized between documents. Evaluators were not given any details about the five translations (such as whether they are human or MT translations or by which MT systems). Every evaluator was assigned only documents that he/she has not yet evaluated in the basic quality + error types evaluations. Guidelines presented to the evaluators are given in Supplementary Methods 1.3.

Evaluators scored 10 consecutive sentences (or the entire document if this was shorter than 10 sentences) from a random section of the document (the same for all five translations), but had access to the source side of the entire document. Every evaluator scored nine different documents. In total, 45 (33 unique) documents and 431 (336 unique) sentences were evaluated by the five evaluators.

When measuring interannotator agreement, in addition to reporting IAA Kappa scores for the evaluation of all five systems (as usual in WMT) in Supplementary Table 9, we also provide IAA Kappa scores for each pair of systems in Supplementary Fig. 12. This confirms the expectation that a higher interannotator agreement is achieved in comparisons of pairs of systems with a large difference in quality.

When carrying out statistical analysis, we first grouped the scores of sentences with multiple evaluations by computing the fluency and adequacy score per sentence and translation from the scores of all evaluators who scored this sentence. Next, we sorted the MT systems by the mean score, using sign test to compare the difference between the consecutive systems (for Fig. 4b). Evaluation of the entire test-set (all originally English sentences) using BLEU for comparison is shown in Supplementary Fig. 13.

### 21 Translation turing test

Participants of the Translation Turing test were unpaid volunteers. The participants were randomly assigned into four non-overlapping groups: A1, A2, B1, B2. Groups A1 and A2 were presented translations by both human reference and CUBBITT. Groups B1 and B2 were presented translations by both human reference and Google Translate (obtained from https://translate.google.cz/ on 13 August 2018). The source sentences in the four groups were identical. Guidelines presented to the evaluators are given in Supplementary Methods 1.4.

The evaluated sentences were taken from originally English part of the WMT18 evaluation test-set (i.e., WMT18-orig-en) and shuffled in a random order. For each source sentence, it was randomly assigned whether Reference translation will be presented to group A1 or A2; the other group was presented this sentence with the translation by CUBBITT. Similarly, for each source sentence, it was randomly assigned whether Reference translation will be presented to group B1 or B2; the other group was presented this sentence with the translation by Google Translate. Every participant was therefore presented human and machine translations approximately in a 1:1 ratio (but this information was intentionally concealed from them).

Each participant encountered each source sentence at most once (i.e., with only one translation), but each source sentence was evaluated for all the three systems. (Reference was evaluated twice, once in the A groups, once in the B groups.) Each participant was presented with 100 sentences. Only participants with more than 90 sentences evaluated were included in our study.

The Translation Turing test was performed as the first evaluation in this study (but after the WMT18 competition) and participants who overlapped with the evaluators of the context-aware evaluations were not shown results from the Turing test before they finished all the evaluations.

In total, 15 participants evaluated a mix of human and CUBBITT translations (five professional translators, six MT researchers, and four other), 16 participants evaluated a mix of human and Google Translate translations (eight professional translators, five MT researchers, and three other). A total of 3081 sentences were evaluated by all participants of the test.

When measuring interannotator agreement, we computed the IAA Kappas (Supplementary Table 10) using our own script, treating the task as a simple binary classification. While in the previous types of evaluations, we computed the IAA Kappa scores using the script from WMT 2016^38^, this was not possible in the Translation Turing test, which does not involve any ranking.

When carrying out statistical analysis, we computed the accuracy for each participant as the percentage of sentences with correctly identified MT or human translations (i.e., number of true positives + true negatives divided by the number of scored sentences) and the significance was assessed using the Fisher test on the contingency table. The resulting *P*-values were corrected for multiple testing with the Benjamini–Hochberg method using matlab function fdr_bh(pValues,0.05,‘dep’,‘yes’)^39^ and participants with the resulting *Q*-value below 0.05 were considered to have significantly distinguished between human and machine translations.

### 22 Block-BT and checkpoint averaging synergy

In this analysis, the four systems from Fig. 2a were compared: block-BT vs mix-BT, both with (Avg) vs without (noAvg) checkpoint averaging. All four systems were trained with a single iteration of backtranslation only, i.e., corresponding to the MT2 system in Supplementary Fig. 2. The WMT13 newstest (3000 sentences) was used to evaluate two properties of the systems over time: translation diversity and generation of novel translations by checkpoint averaging. These properties were analyzed over the time of the training (up to 1 million steps), during which checkpoints were saved every hour (up to 214 checkpoints).

### 23 Overall diversity and novel translation quantification

We first computed the overall diversity as the number of all the different translations produced by the 139 checkpoints between 350,000 and 1,000,000 steps. In particular, for every sentence in WMT13 newstest, the number of unique translations was computed in the hourly checkpoints, separately for block-BT-noAvg and mix-BT-noAvg. Comparing the two systems in every sentence, block-BT-noAvg produced more unique translations in 2334 (78%) sentences; mix-BT-noAvg produced more unique translations in 532 (18%) sentences; and the numbers of unique translations were equal in 134 (4%) sentences.

Next, in the same checkpoints and for every sentence, we compared translations produced by models with and without averaging and computed the number of checkpoints with a *novel*_*Avg∞*_ translation. These are defined as translations that were never produced by the same system without checkpoint averaging (by never we mean in none of the checkpoints between 350,000 and 1,000,000). In total, there were 1801 (60%) sentences with at least one checkpoint with novel_Avg∞_ translation in block-BT and 949 (32%) in mix-BT. When comparing the number of novel_Avg∞_ translations in block-BT vs mix-BT in individual sentences, there were 1644 (55%) sentences with more checkpoints with novel_Avg∞_ translations in block-BT, 184 (6%) in mix-BT, and 1172 (39%) with equal values.

### 24 Diversity and novel translations over time

First, we evaluated development of translation diversity over time using moving window of octuples of checkpoints in the two systems without checkpoint averaging. In particular, for every checkpoint and every sentence, we computed the number of different unique translations in the last eight checkpoints. The average across sentences is shown in Supplementary Fig. 16, separately for block-BT-noAvg and mix-BT-noAvg.

Second, we evaluated development of novel translations by checkpoint averaging over time. In particular, for every checkpoint and every sentence, we evaluated whether the Avg model created a novel_Avg8_ translation, i.e., whether the translation differed from all the translations of the last eight noAvg checkpoints. The percentage of sentences with a novel_Avg8_ translation in the given checkpoint is shown in Fig. 8a, separately for block-BT and mix-BT.

### 25 Effect of novel translations on evaluation by BLEU

We first identified the best model (checkpoint) for each of the systems according to BLEU: checkpoint 775178 in block-BT-Avg (BLEU 28.24), checkpoint 775178 in block-BT-NoAvg (BLEU 27.54), checkpoint 606797 in mix-BT-Avg (BLEU 27.18), and checkpoint 606797 in mix-BT-NoAvg (BLEU 26.92). We note that the Avg and NoAvg systems do not necessarily need to have the same checkpoint with the highest BLEU, however it was nevertheless the case in both block-BT and mix-BT systems here. We next identified which translations in block-BT-Avg and in mix-BT-Avg were novel_Avg8_ (i.e., not seen in the last eight NoAvg checkpoints). There were 988 novel_Avg8_ sentences in block-BT-Avg and 369 in mix-BT-Avg. Finally, we computed BLEU of Avg translations, in which either the novel_Avg8_ translations were replaced with the NoAvg versions (yellow bars in Fig. 8b), or vice versa (orange bars in Fig. 8b); separately for block-BT and mix-BT.

### Reporting summary

Further information on research design is available in the Nature Research Reporting Summary linked to this article.

## Supplementary information

Supplementary Information

Peer Review File

Description of Additional Supplementary Files

Supplementary Data 1

Reporting Summary

## Data Availability

Data used for comparison of human and machine translations may be downloaded at http://hdl.handle.net/11234/1-3209.

## Code availability

The CUBBITT source code is available at https://github.com/tensorflow/tensor2tensor. Codes for analysis of human and machine translations were uploaded together with the analyzed data at http://hdl.handle.net/11234/1-3209.
