# Supplementary material for: Transforming machine translation: a deep learning system reaches news translation quality comparable to human professionals
Source: Nat Commun. 2020 Sep 1;11:4381. doi: 10.1038/s41467-020-18073-9 (PMC7463233; doi:10.1038/s41467-020-18073-9)
Supplement: Supplementary file 1 — Supplementary Information [file 41467_2020_18073_MOESM1_ESM.pdf]

Supplementary Information for

Transforming machine translation: a deep learning system reaches news translation quality comparable to human professionals

Popel et. al. 2020

Correspondence to: popel@ufal.mff.cuni.cz

This PDF file includes:

Supplementary Methods
Supplementary Table 1-10
Supplementary Figure 1-18
Supplementary References

1. Supplementary Methods

1.1. Context-aware evaluation: guidelines

Please evaluate the quality of translations of news articles in the provided Google sheet file. Every line contains one sentence in the original language (English) and two Czech translations. The sentences are structured into documents (i.e., news articles), which are separated by yellow rows. In each document, a section of up to 10 sentences is selected for evaluation (the entire document is shown for the original language, but the cells with gray background should not be edited). Please evaluate the quality of each of the two translations for each sentence using the following values:<br>● **Overall** translation quality: a value between 0 (worst) to 10 (best)<br>● **Adequacy** of the translation: a value between 0 (worst) to 10 (best)<br>○ Adequacy is the degree to which the meaning of the source sentence is preserved in the translation (no information added, no information omitted, no shift of meaning).<br>● **Fluency** of the translation: a value between 0 (worst) to 10 (best)<br>○ Fluency measures how fluent the sentence sounds in the target language (whether the translation is a well-formed natural sentence in the target language, regardless of the source).<br><br>Additionally, please evaluate the **document-level quality** of translation at the end of the evaluated sentences of every document by filling the overall quality, adequacy, and fluency for each of the two translations (these fields are highlighted in red background colour).<br><br>Optionally, you can add a text note into the column “Optional comment” for any row. However, this is really optional; do not spend much time with writing these comments.<br><br>You may consult dictionaries or other sources to check correctness of the translations, but in any case, please **refrain from using any translation systems** for such consultation.<br><br>Try to annotate each document in one go. You can interrupt the annotation after annotating each document. Annotation of each document is an independent task.<br><br>By participating in this annotation, you confirm that you agree with usage of the collected data (after anonymization) for research purposes. We thank you for your time and participation in this research!
Guidelines presented to the evaluators before they started the translation quality evaluation.

1.2. Error type evaluation: guidelines

You will be presented with the file you have already annotated for adequacy, fluency and overall translation quality. In this additional annotation, your task is to classify each translation error into one of the following eight error types:<br>● **Addition** of meaning<br>● **Omission** of meaning<br>● Wrong translation of an **ambiguous** word (i.e. a word with multiple meanings in the source language was translated poorly)<br>● A **shift** of meaning (not based on ambiguous words)<br>● **Other adequacy** error<br>● **Grammar** error<br>● **Spelling** error (including typos, excluding grammar errors)<br>● **Other fluency** error (including wrong word order)<br>Please enter the number of errors (1,2,3,...) of a given type in each translation into the corresponding column. If there is no error of a given type, you can leave the corresponding column empty.<br><br>We would like to ask you to read also translations that you scored with 10 and double-check that they do not contain any of the error types listed above.<br><br>Optionally, you can edit any of your previously filled scores of adequacy, fluency and overall translation quality. You can also add additional comments into the *Optional comment* column.<br><br>In addition to the eight error types, please indicate whether the translation was wrong because of cross-sentence context (i.e., because of other sentences in the document) by filling the number of errors due to cross-sentence context into the column "**Context**". This can be, for example:<br>● using a wrong translation of an ambiguous word with respect to the context of the document (given the knowledge from a different sentence, the meaning of an ambiguous word should have been clear here),<br>● poorly fluent connection of consecutive sentences in the translation in contrast to the connection expressed in the source language,<br>● wrong gender of a person/place (where the gender cannot be resolved within the sentence but is known from the context of the document), etc.<br><br>Optionally, you can mark incorrect parts of the translation with a colour of your choice. However, this is really optional; do not spend much time on the colouring.
Guidelines presented to the evaluators before they started the evaluation or error types.

1.3. Evaluation of five MT systems: guidelines

Please evaluate the quality of translations of news articles in the provided Google sheet file. Every line contains one sentence in the original language (English) and five Czech translations. The sentences are structured into documents (i.e., news articles), which are separated by yellow rows. In each document, a section of up to 10 sentences is selected for evaluation (the entire document is shown for the original language, but the cells with gray background should not be edited). Please evaluate the quality of each of the five translations for each sentence using the following values:<br>● **Adequacy** of the translation: a value between 0 (worst) to 10 (best)<br>○ Adequacy is the degree to which the meaning of the source sentence is preserved in the translation (no information added, no information omitted, no shift of meaning).<br>● **Fluency** of the translation: a value between 0 (worst) to 10 (best)<br>○ Fluency measures how fluent the sentence sounds in the target language (whether the translation is a well-formed natural sentence in the target language, regardless of the source).<br><br>You can use **scores with one decimal place** (0.1, 0.2,…9.9) for both adequacy and fluency in order to distinguish between small differences in quality of the five translations.<br><br>Optionally, you can add a text note into the column "Optional comment" for any row. However, this is really optional; do not spend much time with writing these comments.<br><br>You may consult dictionaries or other sources to check correctness of the translations, but in any case, please refrain from using any translation systems for such consultation.<br><br>Try to annotate each document in one go. You can interrupt the annotation after annotating each document. Annotation of each document is an independent task.<br><br>By participating in this annotation, you confirm that you agree with usage of the collected data (after anonymization) for research purposes. We thank you for your time and participation in this research!
Guidelines presented to the evaluators before they started the evaluation of five MT systems.

1.4. Translation Turing Test: Guidelines

For this "Translation Turing test", we need Czech native speakers fluent in English. Select one of the following three options:<br>● I am an MT researcher (I have experience with machine translation research).<br>● I am either a professional English-Czech translator or I have a degree in British or American studies or Translation studies.<br>● Other.<br><br>The attached table contains 100 independent sentences, each translated either by a human translator, or by a machine translation system.<br>● Fill the column "Is human" for each sentence in the attached document:<br>○ Fill 1, if you think that the translation was performed by a human translator.<br>○ Fill 0, if you think that the translation was performed by a machine translation.<br>○ (Any other values are not allowed.)<br>● Optionally, you can add a text note into the column "Optional comment". However, this is really optional; do not spend much time with writing these comments.<br>● The document can contain any ratio of human vs machine translations, so trying to "balance" your guesses between humans and machines is not advisable.<br>● You may consult dictionaries or other sources to check correctness of the translations, but it any case, please **refrain from using any translation systems** for such consultation. (Again, you are not required to do this at all, do not spend time on this research. It is sufficient to guess the origin of the translation.)<br>● In addition, you can optionally include information about your age and approximately how many years have you been learning English.<br><br>By completing and returning the questionnaire, you confirm that you agree with anonymous usage of the collected data for research purposes. We thank you for your time and participation in this research!
Guidelines presented to the participants before they started the Translation Turing test.

2. Supplementary Figures

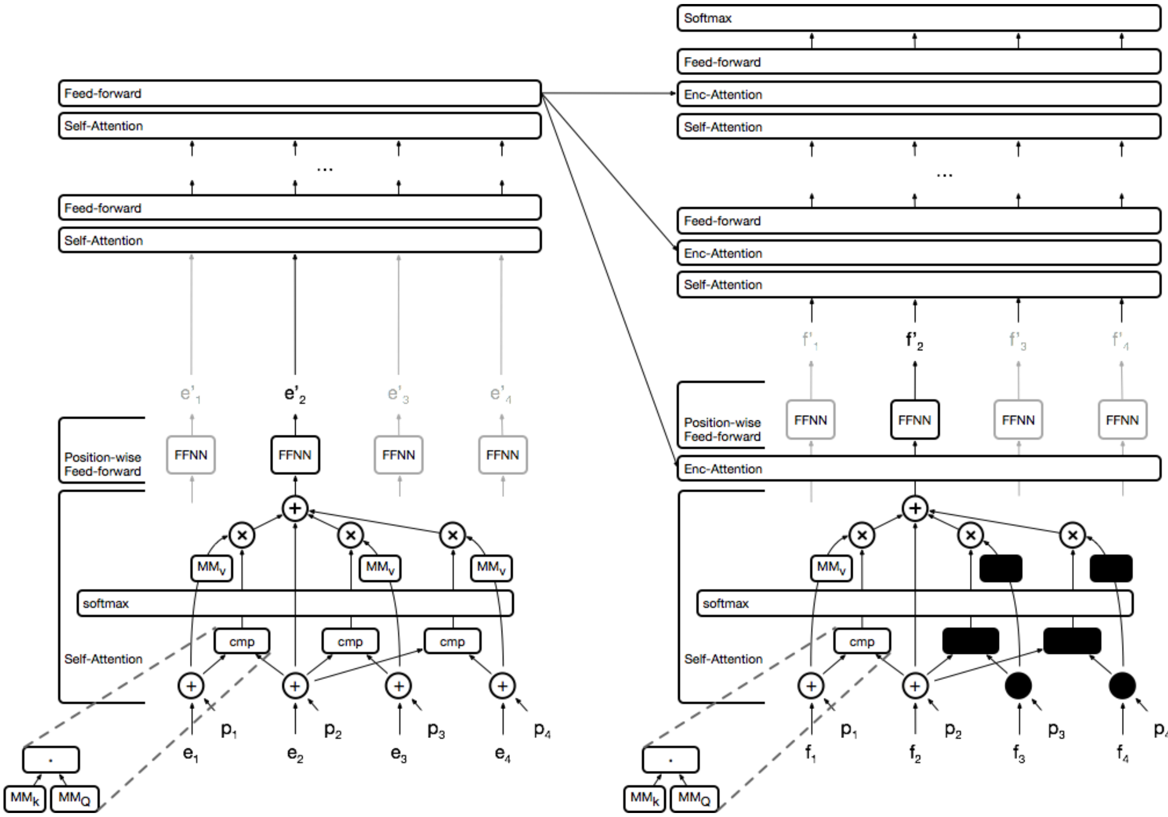

Supplementary Figure 1. Transformer architecture. The bottom part represents the attention layer, computing similarity (cmp) based on a dot-product between the keys and the queries. The results are passed through softmax and these attention weights are multiplied with the values to create the output of the self-attention layer. These outputs are passed through position-wise feed-forward layers and the whole stack is repeated multiple times, both in the encoder and decoder part of the model. FFNN = feed forward neural network, MM = matrix multiplication.

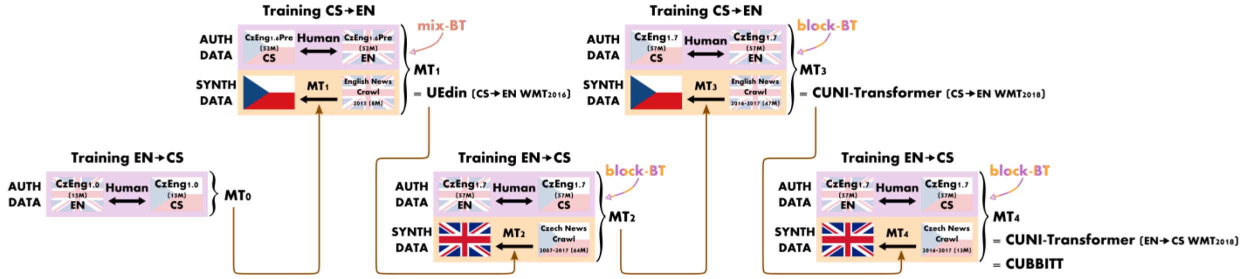

Supplementary Figure 2. Diagram of how English-Czech CUBBITT was trained. System MT0 was EN→CS UEdin model trained with authentic parallel data CzEng 1.0[1]. System MT1 (winner of CS→EN WMT16[2]) was CS→EN UEdin model trained with mix-BT using monolingual EN data translated by MT1 (i.e., synthetic data). System MT2 was EN→CS Transformer model trained with block-BT using monolingual CS data translated by MT1. System MT3 was CS→EN Transformer model trained with block-BT using monolingual EN data translated by MT2. Finally, system MT4 was EN→CS Transformer model trained with block-BT using monolingual CS data translated by MT3. This final system MT4 is the CUBBITT system and also the winner of EN→CS WMT18, submitted under the name CUNI-Transformer.

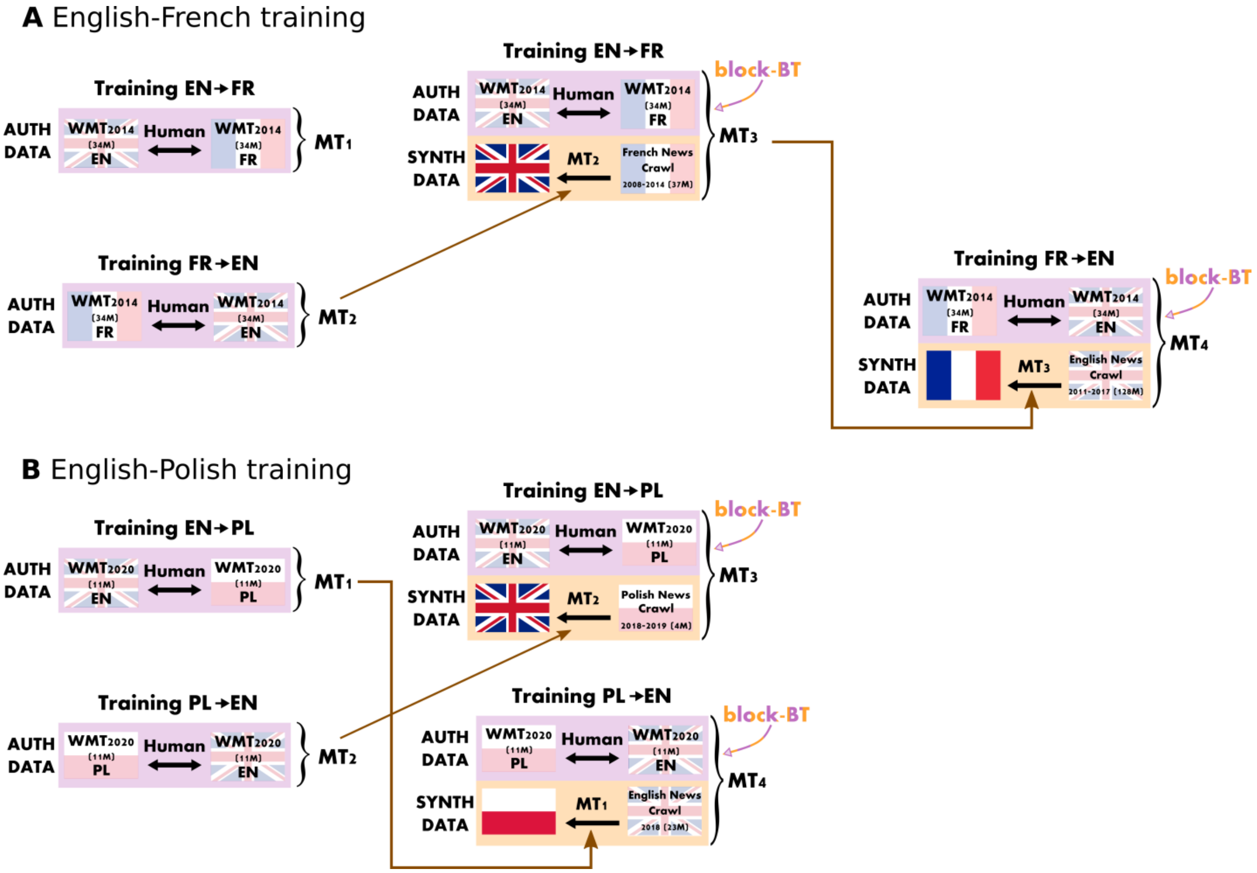

Supplementary Figure 3 Diagram of how CUBBITT was trained for other language pairs. Language pairs shown are English-French (A) and English-Polish (B). When training the English-French MT3 system, we tried both starting the training from scratch (i.e. standard random initialization) and initializing the parameters with the English-French MT1 system; both experiments resulted in similar final BLEU (the latter converged faster, as expected).

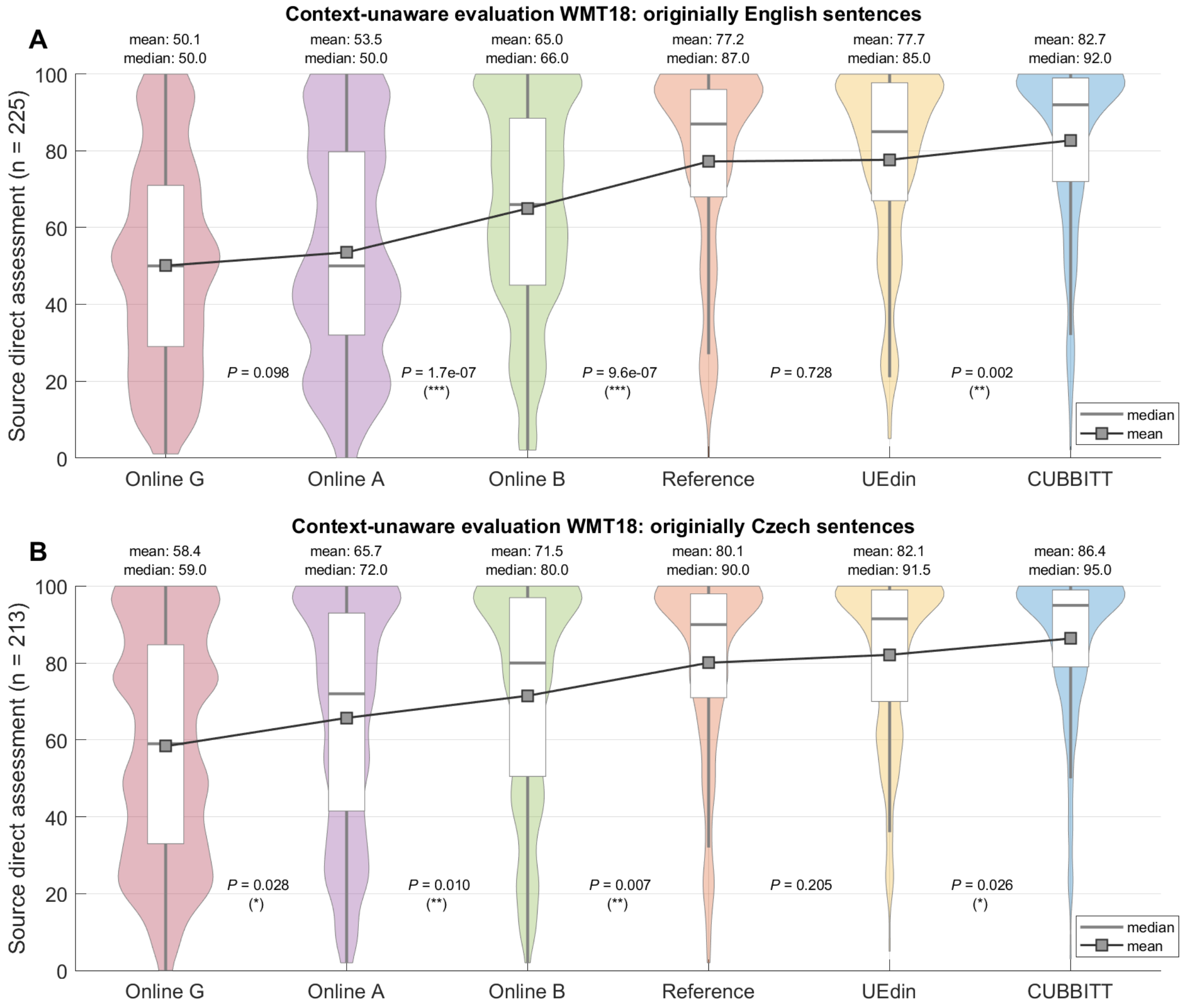

Supplementary Figure 4. CUBBITT outperformed UEdin and human reference in both originally English and originally Czech sentences in WMT18 source direct assessment. Results from context-unaware evaluation in WMT18, showing distributions of source direct assessment (SrcDA) of five MT systems and human reference translation, sorted by average score. CUBBITT was submitted under the name CUNI-Transformer. Online G, A, and B are three anonymised online MT systems. Sign test was used to assess differences between the systems. A: Originally English source sentences only. B: Originally Czech source sentences only.

Source	Translation1	T1_overall	T1_adequacy	T1_fluency	Translation2	T2_overall	T2_adequacy	T2_fluency	Optional comment
Kabul convoy attack: Nato soldier killed in suicide blast									
A suicide bomber has attacked a military convoy in the Afghan capital, Kabul, killing a Nato soldier and wounding several others, officials say.									
Five soldiers and an interpreter were injured, a Nato statement said.									
Unconfirmed local reports say three civilians also died in the incident in the city's Qarabagh district.									
The bombing is the latest violence to have hit Afghanistan this month - on Tuesday more than 30 people were killed in a mosque blast in Herat.	Bombový útok je posledním násilím, které tento měsíc zasáhlo Afghánistán – v úterý bylo při výbuchu mešity v Herátu zabito více než 30 lidí.	8	9	7	Tento měsíc zasáhl Afghánistán bombový útok - v úterý bylo zabito více než 30 lidí při výbuchu v herátské mešitě.	7	7	8	T1 poněkud kostrbaté; T2 jako by nebral v úvahu kohezi textu.
On Monday a suicide bomber killed two people near the Iraqi embassy in Kabul.	V pondělí sebevražedný atentátník zabil dva lidi poblíž irácké ambasády v Kábulu.	10	10	10	V pondělí zabil sebevražedný útočník dva lidi poblíž irácké ambasády v Kábulu.	10	10	10	
A Nato statement said that Thursday evening's blast happened when a joint patrol with the Afghan army was attacked by a "personnel-borne improvised explosive device."	V prohlášení NATO se uvádí, že k výbuchu došlo ve čtvrtek večer, když byla společná hlídka s afghánskou armádou napadena „improvizovaným výbušným zařízením neseným personálem".	8	8	8	NATO ve svém vyjádření uvedlo, že k úternímu výbuchu došlo poté, co byla hlídka spolu se členy afghánské armády napadena „místní osobou s podomácku vyrobeným výbušným zařízením".	8	7	9	T2: "úterní výbuch"
The statement said that all those injured are in a stable condition and are being treated at the US military hospital at Bagram airfield.	V prohlášení se uvádí, že všichni zranění jsou ve stabilizovaném stavu a jsou ošetřováni v americké vojenské nemocnici na letišti Bagram.	8	9	7	V prohlášení se dále uvádí, že všichni zranění vojáci jsou mimo ohrožení života a nacházejí se v americké vojenské nemocnici na letecké základně Bagrám.	9	8	10	T2 ok, až na to, že dělá ze všech raněných vojáky, a pokud jde o jejich zdravotní stav, je možná až moc optimistický
Correspondents say the growing strength of the Taliban and the group known as so-called Islamic State (IS) in Qarabagh is a major source of concern to Nato forces based in nearby Bagram.	Podle korespondentů je rostoucí síla Talibanu a skupiny známé jako tzv. Islámský stát (IS) v Qarabaghu hlavním zdrojem obav sil NATO se sídlem v nedalekém Bagrámu.	10	10	9	Podle vyjádření zpravodajů je narůstající moc Tálibánu a skupiny známé jako Islámský stát (IS) v oblasti Qarabagh významným zdrojem obav spojeneckých sil NATO v nedalekém Bagrámu.	10	10	10	v obou českých překladech mi vadí doslovnost struktury "je zdrojem obav sil NATO", nicméně do bodování jsem nezahrnula (upřednostnila bych něco na způsob "Podle... se velení jednotek NATO se sídlem v nedalekém B. obává rostoucí...."
According to the United Nations, Afghanistan saw at least 1,662 civilian deaths in the first half of 2017, with about 20% of those in the capital.	Podle Organizace spojených národů si Afghánistán v první polovině roku 2017 vyžádal nejméně 1 662 civilních obětí, z toho asi 20% v hlavním městě.	8	10	9	Podle OSN zemřelo v Afghánistánu v první polovině roku 2017 minimálně 1662 civilistů, z čehož 20 % obětí zemřelo v hlavním městě.	8	10	8	
At the end of July, a suicide car bomb killed at least 30 people in a mainly Shia district of Kabul.	Na konci července sebevražedná bomba v autě zabila nejméně 30 lidí v převážně šíitské čtvrti Kábulu.	9	10	8	Na konci července usmrtila bomba umístěná v automobilu alespoň 30 lidí v kábulské čtvrti Shia.	8	7	9	T2: "alespoň", kábuská čtvrť Shia
The Taliban said they had carried out that attack.	Taliban řekl, že ten útok provedl.	5	10	5	K útoku se přihlásil Tálibán.	10	10	10	
On 31 May, a huge bombing in the centre of the city killed more than 150 people, the deadliest militant attack in the country since US-led forces ousted the Taliban from power in 2001.	Dne 31. května při obrovském bombovém útoku v centru města zahynulo více než 150 lidí, což byl nejnebezpečnější útok ozbrojenců v zemi od roku 2001, kdy jednotky vedené Spojenými státy odstavily Taliban od moci.	7	8	7	31. května zabil velký výbuch v centru města více než 150 lidí. Jedná se o nejsmrtelnější militantní útok v zemi od té doby, co americké jednotky svrhly vládu Tálibánu v roce 2001.	8	9	9	T1: "...což byl"; "nejnebezpečnější" T2: "militantní" + slovosl. (v r. 2001)
The violence underlines the precarious security situation in Afghanistan as US President Donald Trump weighs up whether to increase the number of US troops aiding the military and police in the country.	Násilí podtrhuje nejistou bezpečnostní situaci v Afghánistánu, když americký prezident Donald Trump zvažuje, zda zvýšit počet amerických vojáků, kteří pomáhají armádě a policii v zemi.	8	8	7	Násilí podkopává již tak pochybnou situaci v Afghánistánu a prezident USA Donald Trump zvažuje navýšení počtu amerických vojáků, kteří by měli pomáhat vojenským jednotkám a policistům v zemi.	8	8	9	T1: "když" T2: "podkopává pochybnou situaci"

Supplementary Figure 5. Example evaluation sheet. The columns T1_overall, T1_adequacy, T1_fluency, T2_overall, T2_adequacy, T2_fluency, and Optional comment were originally empty and filled by the evaluator.

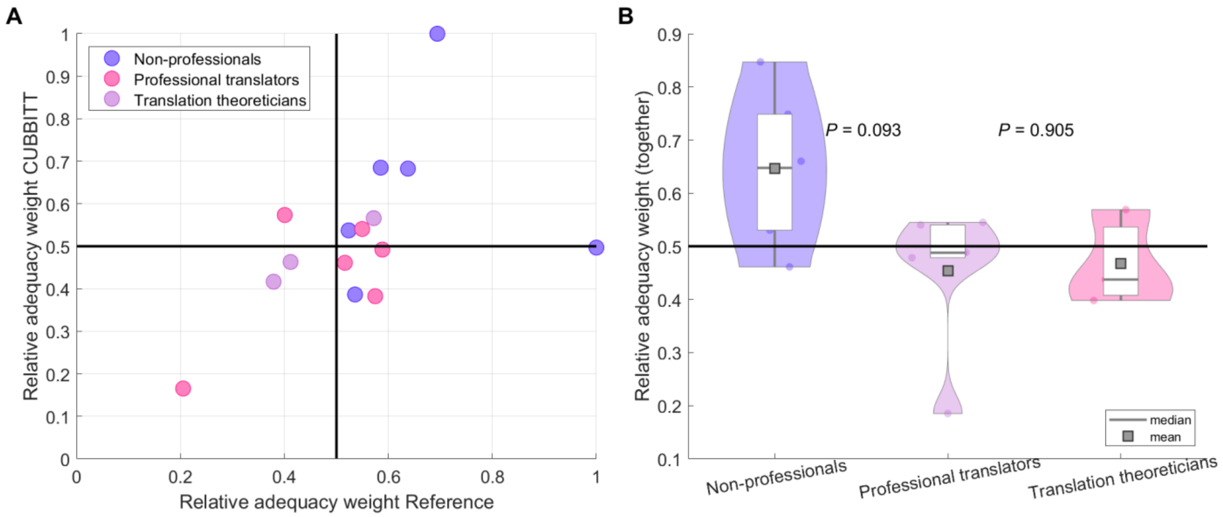

Supplementary Figure 6. Relative contribution of adequacy and fluency in the overall score. A: The relative contribution of adequacy for human reference translations (x-axis) and CUBBITT translations (y-axis) in individual evaluators. The relative contributions were computed by fitting a linear model of weighting adequacy and fluency in the overall quality and the relative weight of adequacy is shown in the figure. Values below 0.5 represent higher contribution of fluency compared to adequacy, while values above 0.5 represent higher values of adequacy. B: Distributions of the relative weights (an average of values on x-axis and y-axis from (A)) shown for the three groups of evaluators. Mann–Whitney U (ranksum) test was used to assess differences between the groups (however, the numbers of evaluators are low).

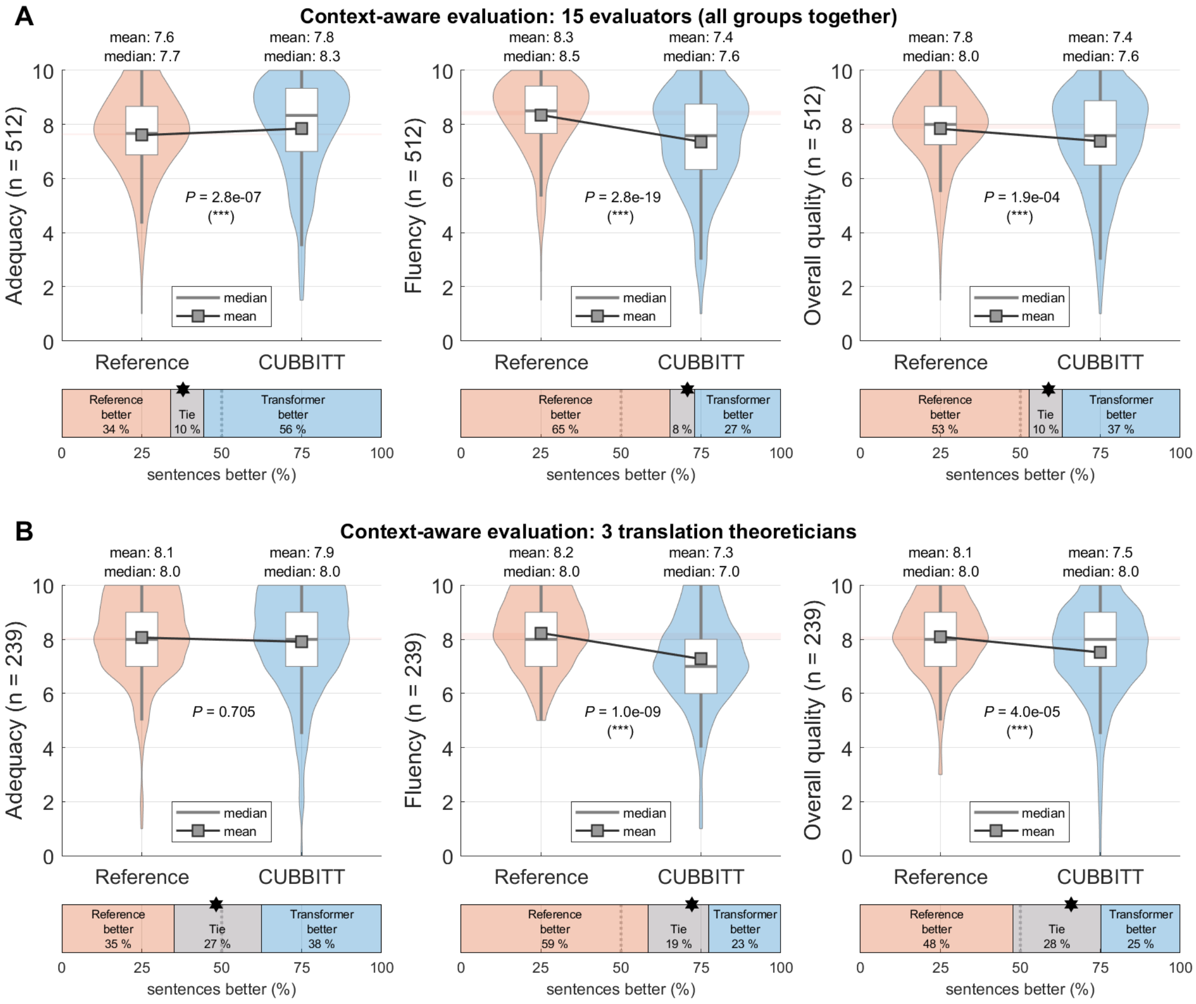

Supplementary Figure 7. Context-aware evaluation of translation quality of CUBBITT compared to human reference. A: Results of the context-aware evaluation for the entire cohort of all 15 evaluators (512 unique sentences). The scores (0-10) are shown as violin plots with boxplots, while the boxes below represent the percentage of sentences scored better in reference (orange), Transformer (blue), or the same (gray); the star symbol marks the ratio of orange vs. blue ignoring gray. Sign test was used to evaluate difference between human and machine translation. B: As in (A), but showing only evaluations of the exploratory (underpowered) group of three translation theoreticians on 239 sentences.

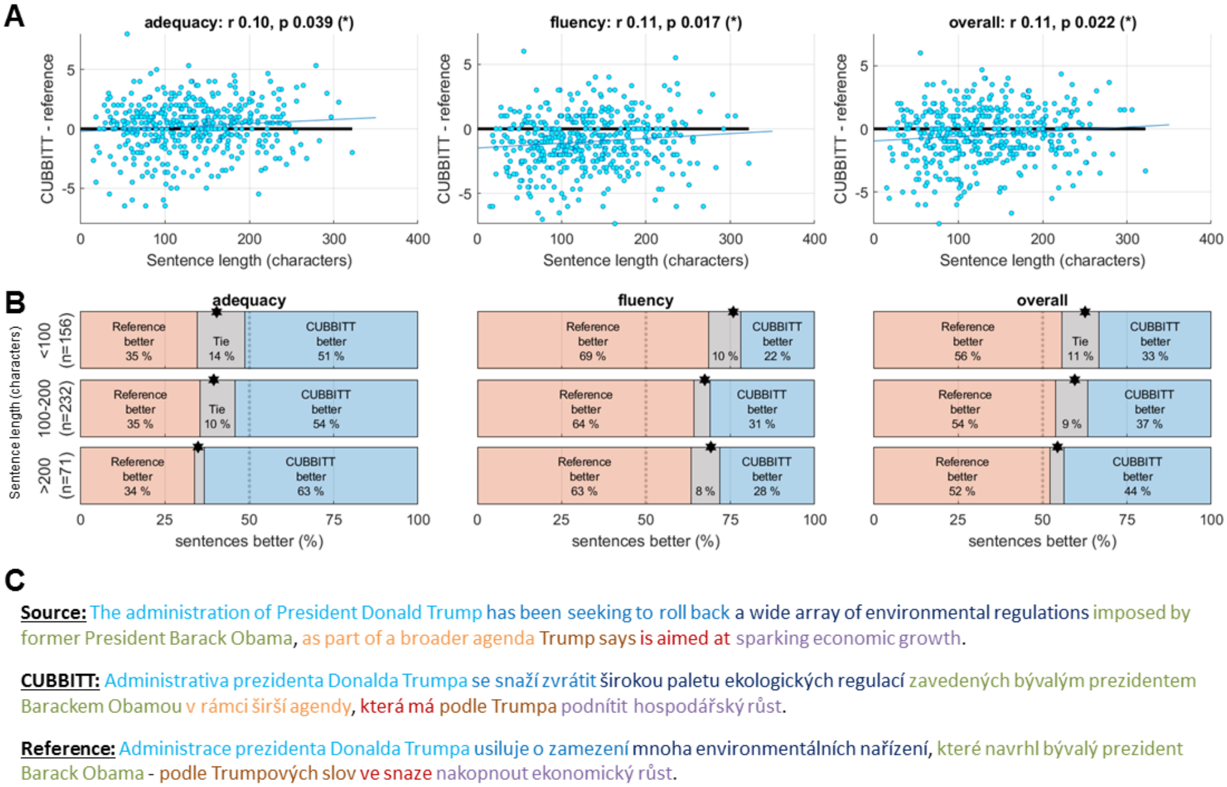

Supplementary Figure 8. The relationship between sentence length and translation quality. A: The correlation between the length of the source sentence (number of characters) and the evaluation of quality of the difference between CUBBITT and human reference is shown separately for adequacy (left), fluency (middle), and overall quality (right). The blue line represents a linear fit through the individual data points (sentences). The values of Pearson *r* and *p*-value of the correlation are shown above the graphs. B: The boxes represent the percentage of sentences that scored better in reference (orange), CUBBITT (blue), or the same (gray); the star symbol marks the ratio of orange vs. blue, ignoring gray. This analysis is shown separately for short sentences (top row, less than 100 characters, 156 sentences), medium-length sentences (middle row, 100-200 characters, 232 sentences), and long sentences (bottom row, more than 200 characters, 71 sentences). C: An example of a long sentence (235 characters), translated well by CUBBITT (adequacy 10, fluency 9, overall quality 9).

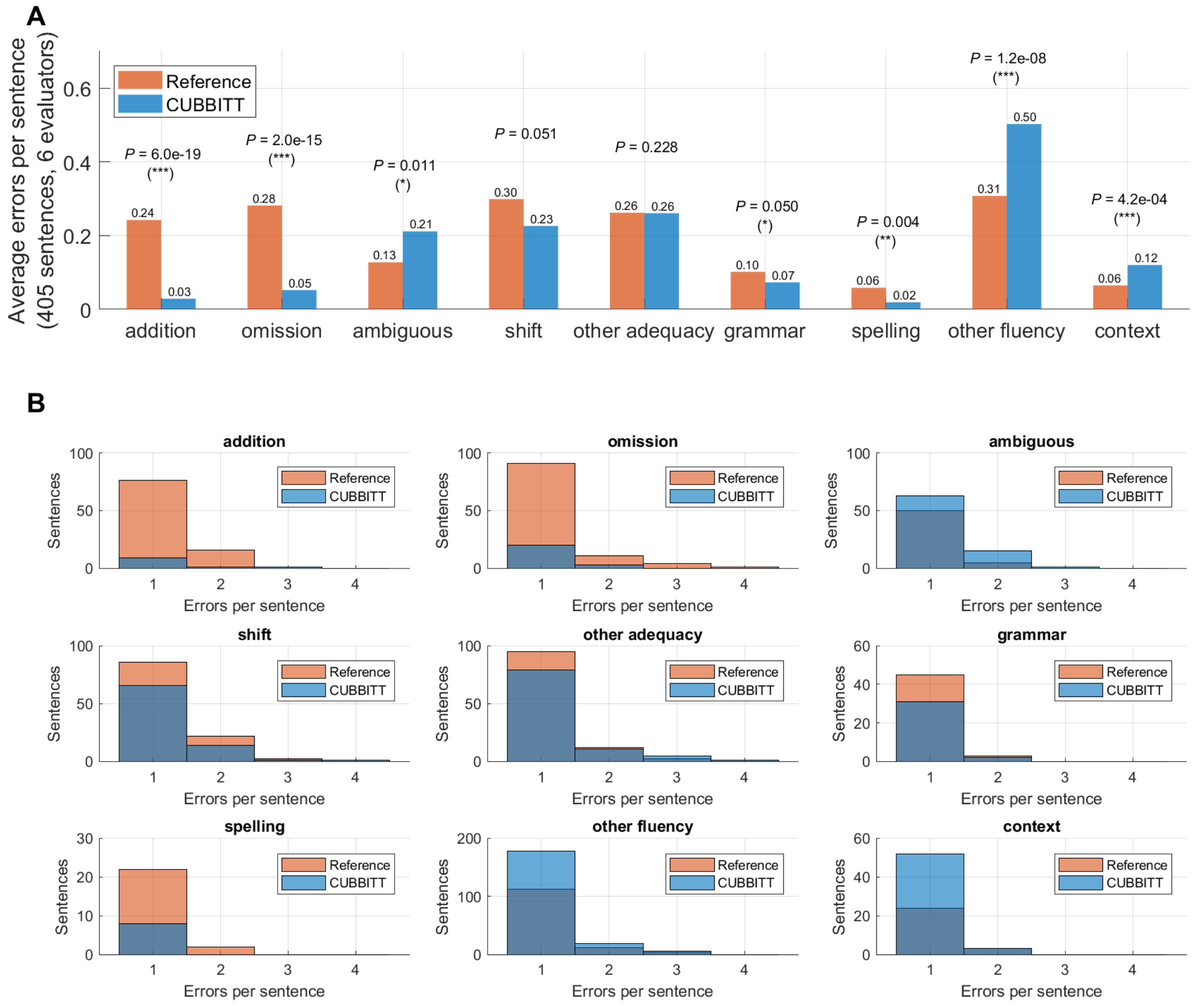

Supplementary Figure 9. Context-aware evaluation of error types made by CUBBITT and human reference translations. A: Average number of errors per sentence is shown for translations by human reference and CUBBITT. Errors in 405 sentences were evaluated by six evaluators (three professional translators and three non-professionals). Sign test was used to evaluate difference between human and machine translation. B: Distribution of the number of errors per sentence shown as a histogram for human reference translations (orange) and CUBBITT translation (blue).

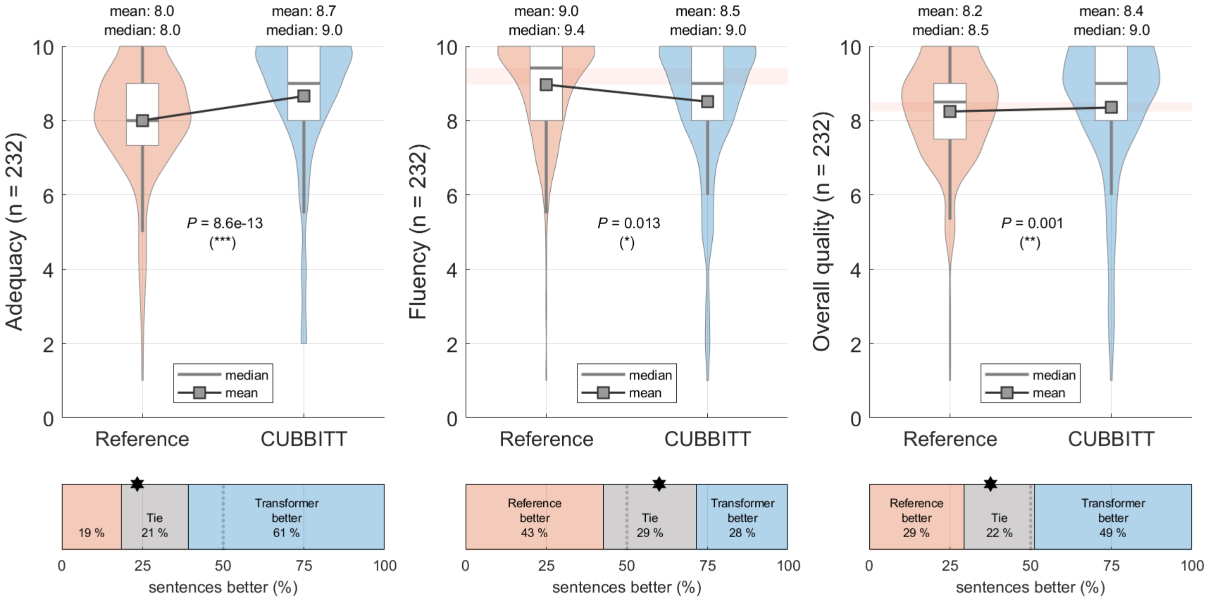

Supplementary Figure 10. Results from context-aware evaluation in non-professionals on sentences without context errors. Results of adequacy, fluency, and overall quality scores shown only for sentences without a context error in the human reference and CUBBITT translation (according to the error-type evaluation). Sign test was used to evaluate difference between human and machine translation.

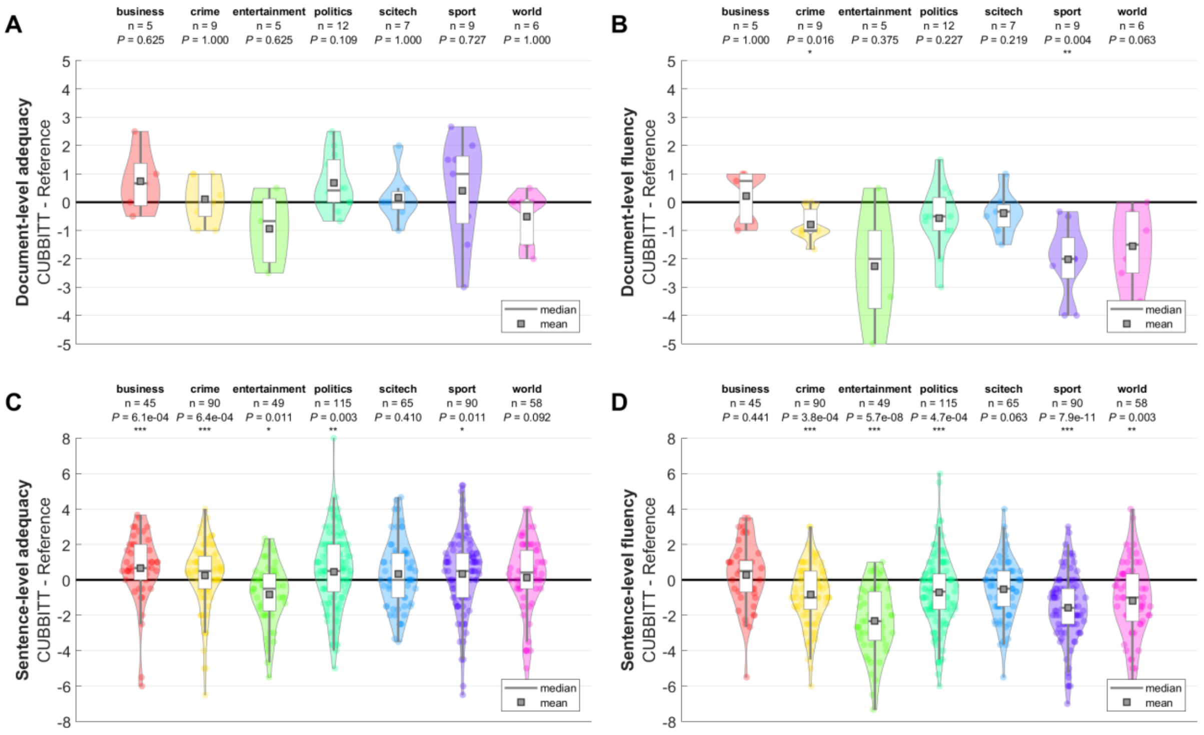

Supplementary Figure 11. Context-aware evaluation of translation quality with respect to document classes. A-B: document-level evaluation for the seven types of documents shown as distribution of the difference of human reference translation score - CUBBITT translation score, shown separately for adequacy (A) and fluency (B). C-D: Sentence-level results for adequacy (C) and fluency (D). Sign test was used to assess differences between the human reference and CUBBITT translations.

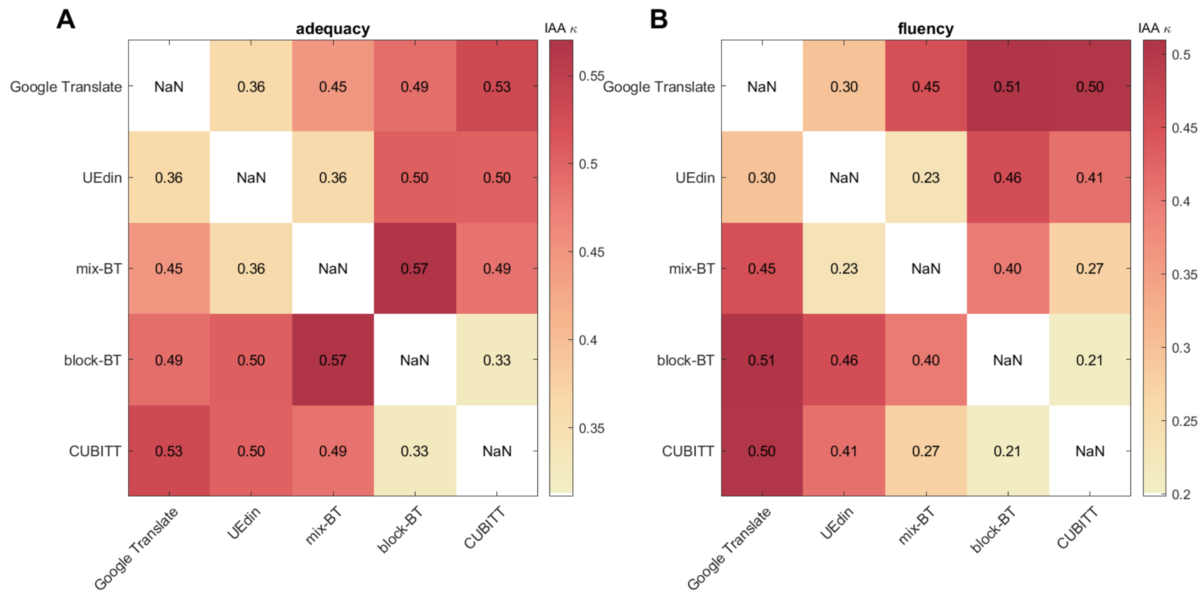

Supplementary Figure 12. IAA Kappa scores for the evaluation of five MT systems. A: adequacy. B: fluency. The Kappa values were computed in the same way as in WMT 2012–2016[3].

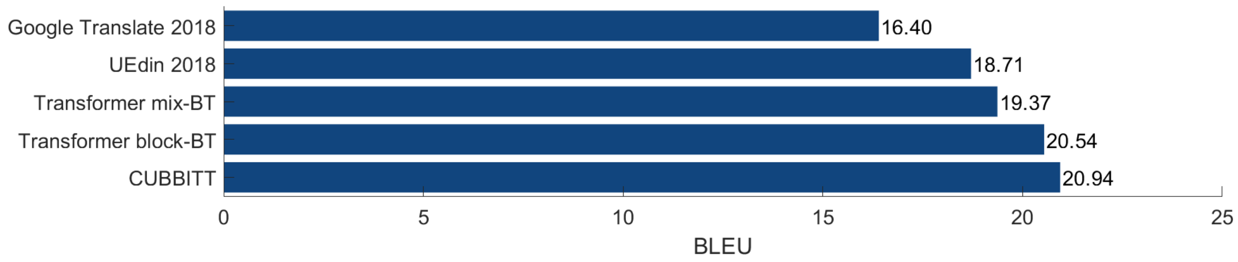

Supplementary Figure 13. BLEU scores of the translations in the evaluation of five MT systems. All originally English sentences from the WMT18 English→Czech news test set were used to compute BLEU scores by comparing the five MT systems to human reference translations.

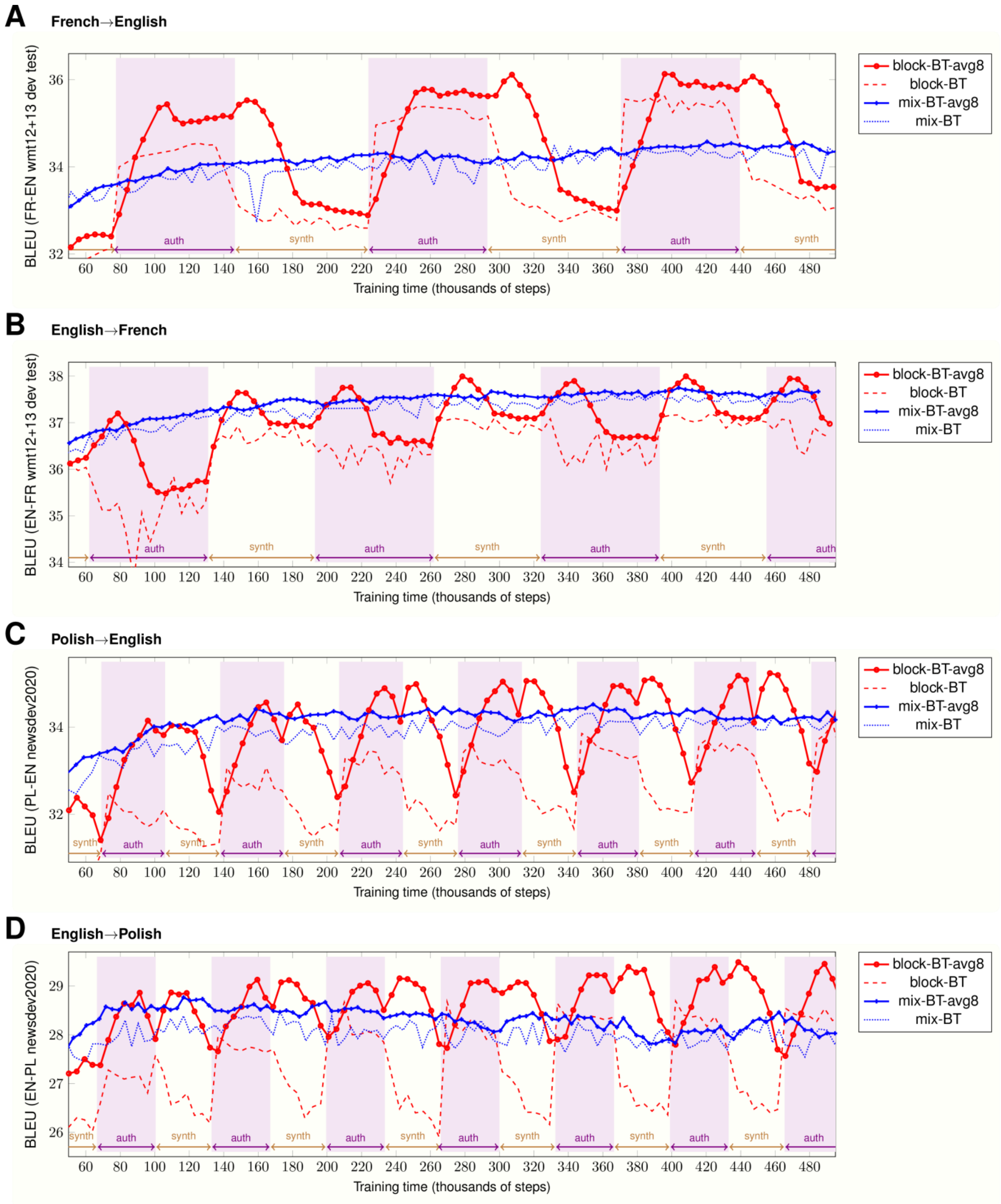

Supplementary Figure 14. Block-BT with checkpoint averaging improves BLEU over mix-BT also for other language pairs. A: French→English, B: English→French, C: Polish→English, D: English→Polish. The data are consistent with the behavior of the systems on the English-Czech language pair (Fig. 2) in that it shows major benefit of block-BT from checkpoint averaging, outperforming mix-BT with checkpoint averaging.

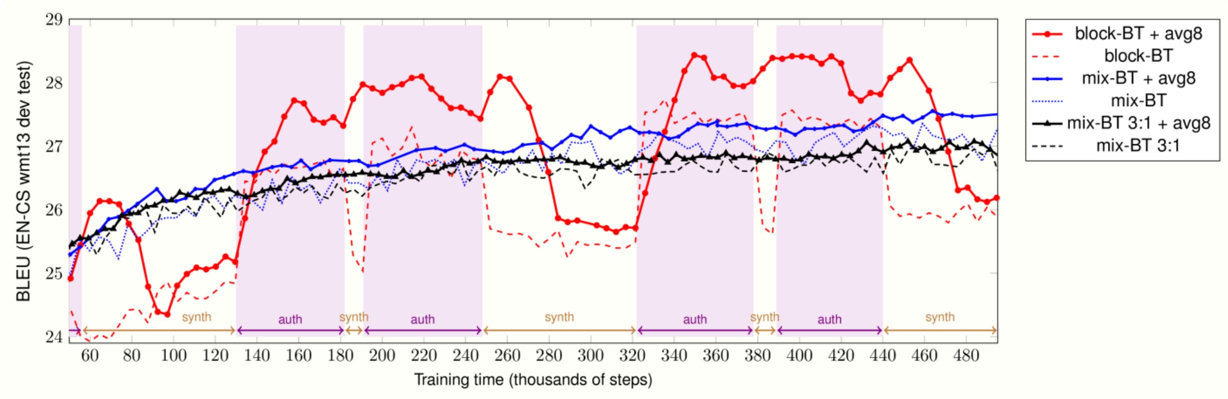

Supplementary Figure 15. Ratio of authentic and synthetic data does not explain the improved BLEU via block-BT with checkpoint averaging. One possible explanation of the benefits brought by block-BT and checkpoint averaging is that the averaging window explores multiple ratios of networks trained on synthetic and authentic data, picking the optimal one. When averaging 8 checkpoints, the peak of CUBBITT's performance was reached when six networks trained on authentic data and two networks trained on synthetic data were averaged (i.e., 3:1 ratio). The ratio of authentic to synthetic data in mix-BT (shown in blue) was approximately 1.2:1 in total and therefore also in every checkpoint. We note that the total amount of data presented to block-BT during training had the same ratio, but the block regime allows for local weighting of networks trained on authentic and synthetic data with different ratios. To test the importance of the data ratio, we repeated the training of mix-BT network on data where authentic data were overrepresented over synthetic data in approximately 3:1 ratio (shown in black). The resulting curves demonstrate that such a network does not bring any advantage compared to the original mix-BT.

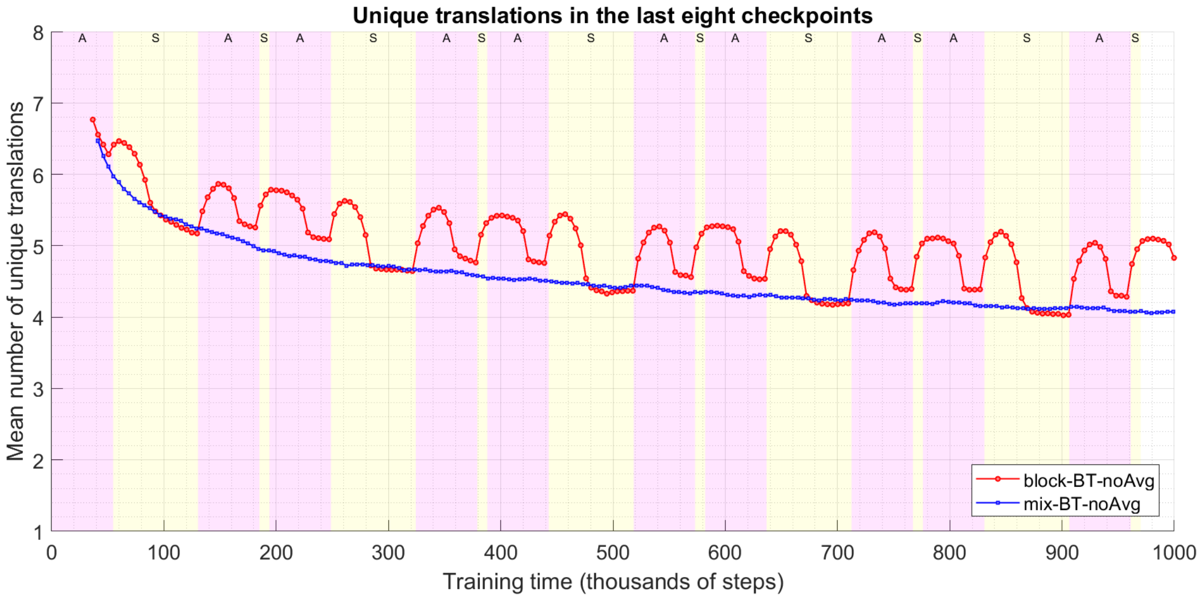

Supplementary Figure 16. Diversity of translations in block-BT and mix-BT. The number of unique translations in the preceding eight checkpoints, averaged over the sentences in WMT13 newstest, are shown over time, separately for block-BT (red) and mix-BT (blue), both models without checkpoint averaging. The checkpoints trained in AUTH blocks are denoted by magenta background and letter A, while the SYNTH blocks are shown in yellow background and letter S.

Source While cross-examining Manning at a pre-trial hearing at Ft. Meade, Maryland, prosecutor Maj. Ashden Fein asserted that records of weekly visits Manning had with unit officers during nine months of detention at Quantico, Virginia, show no complaints about his treatment.

block-BT-Avg Při křížovém výslechu Manninga při předběžném slyšení ve Ft. Meade v Marylandu státní zástupce major Ashden Fein tvrdil, že záznamy z týdenních návštěv Manninga s důstojníky jednotky během devíti měsíců vazby v Quanticu ve Virginii neukazují žádné stížnosti na jeho zacházení.

C1: AUTH Při křížovém výslechu Manninga během předběžného slyšení ve věznici Ft. Meade v Marylandu prokurátor major Ashden Fein tvrdil, že záznamy z týdenních návštěv, které Manning s důstojníky během devíti měsíců zadržení v Quanticu ve Virginii absolvoval, neukazují žádné stížnosti na jeho léčbu.

C2: AUTH Během křížového výslechu Manninga během předběžného slyšení ve Ft. Meade ve státě Maryland žalobce Maj. Ashden Fein tvrdil, že záznamy o týdenních návštěvách Manninga s důstojníky během devíti měsíců zadržení v Quanticu ve Virginii neukazují žádné stížnosti na jeho léčbu.

C3: AUTH Při křížovém výslechu Manninga během předběžného slyšení ve Ft. Meade v Marylandu státní zástupce major Ashden Fein tvrdil, že záznamy týdenních návštěv, které Manning absolvoval s důstojníky jednotky během devíti měsíců vazby v Quanticu ve Virginii, neukazují žádné stížnosti na jeho léčbu.

C4: AUTH Při výslechu Manninga během předběžného slyšení ve Ft. Meade ve státě Maryland státní zástupce major Ashden Fein tvrdil, že záznamy z týdenních návštěv Manninga s důstojníky jednotky během devíti měsíců vazby v Quanticu ve Virginii neukazují žádné stížnosti na jeho léčbu.

C5: AUTH Při křížovém výslechu Manninga během předběžného slyšení ve Ft Meade v Marylandu státní zástupce major Ashden Fein tvrdil, že záznamy z týdenních návštěv Manninga s důstojníky jednotky během devíti měsíců vazby v Quanticu ve Virginii neukazují žádné stížnosti na jeho léčbu.

C6: AUTH Během křížového výslechu Manninga při předběžném slyšení ve Ft Meade v Marylandu státní zástupce major Ashden Fein tvrdil, že záznamy o týdenních návštěvách Manninga s důstojníky jednotky během devíti měsíců vazby v Quanticu ve Virginii neukazují žádné stížnosti na jeho léčbu.

C7: SYNTH Zatímco křížový výslech Manninga při předběžném slyšení ve Ft. Meade v Marylandu, prokurátor Maj. Ashden Fein tvrdil, že záznamy týdenních návštěv Manninga s jednotkou důstojníků během devíti měsíců zadržení v Quanticu ve Virginii, neukazují žádné stížnosti na jeho zacházení.

C8: SYNTH Zatímco křížový výslech Manningové při přípravném jednání ve Ft. Meade ve státě Maryland, prokurátor Maj. Ashden Fein tvrdil, že záznamy týdenních návštěv Manningové měly s jednotkami důstojníků během devíti měsíců zadržení v Quanticu ve Virginii, neukazují žádné stížnosti na jeho zacházení.

Supplementary Figure 17. An example of novelAvg translation by crossover from authentic and synthetic blocks in checkpoint averaging. The figure first shows the source English sentence, the translation by block-BT with checkpoint averaging (block-BT-Avg, checkpoint 775178), and translations by the eight preceding noAvg checkpoints that were averaged (six trained in authentic blocks, two in synthetic blocks). The sentence has three elements highlighted, all well translated by block-BT-Avg. However, in none of the checkpoints without averaging are all three elements correctly translated. The first two are correctly translated only in the AUTH-trained checkpoints, while the last one is correctly translated only in the SYNTH-trained checkpoints. In spite of the last element being correctly translated only in two of the eight averaged checkpoints, it is correctly used in the averaged model.

A

Source sentence: "He was an original **guy** and lived life to the full" said **Gray** in a statement.

SYNTH: **chlap** AUTH: **člověk** SYNTH: **Šedivý** AUTH: **Gray**

Translation of block-BT-Avg: "Byl to originální chlap a žil život naplno," uvedl Gray v prohlášení.

Checkpoint 1 (SYNTH): "Byl to originální chlap a žil život naplno" uvedl **Šedivý** v prohlášení.
Checkpoint 2 (SYNTH): "Byl to originální chlap a žil život naplno" uvedl **Šedivý** v prohlášení.
Checkpoint 3 (AUTH): „Byl to originální **člověk** a žil život naplno," uvedl Gray v prohlášení.
Checkpoint 4 (AUTH): „Byl to originální **člověk** a žil život naplno," uvedl Gray v prohlášení.
Checkpoint 5 (AUTH): „Byl to originální **člověk** a žil život naplno," uvedl Gray v prohlášení.
Checkpoint 6 (AUTH): „Byl to originální **člověk** a žil život naplno," uvedl Gray v prohlášení.
Checkpoint 7 (AUTH): „Byl to originální **člověk** a žil život naplno," uvedl Gray v prohlášení.
Checkpoint 8 (AUTH): „Byl to originální **člověk** a žil život naplno," uvedl Gray v prohlášení.

B

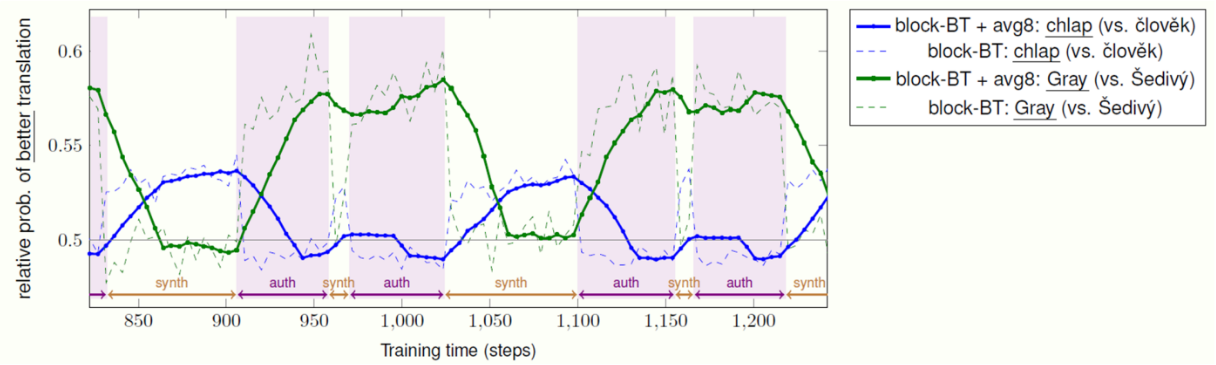

Supplementary Figure 18. Checkpoint averaging takes into account the confidence of averaged networks. We note an important feature of checkpoint averaging, which is that it does much more than simply pick the most frequent translation of each phrase in the eight noAvg-checkpoint translations. This can be illustrated by the example shown above, translated well by block-BT-Avg (A). Inspecting translations of the word "guy" in different checkpoints, we observed a consistent pattern that SYNTH-trained networks prefer "chlap", but AUTH-trained networks prefer "člověk". Interestingly, throughout the training, as few as 1 or 2 SYNTH-trained networks in the averaging window (8 networks) were sufficient to push through the translation preferred by SYNTH networks (which is the more appropriate one, as it reflects the informality evident from the article context). On the other hand, some of the SYNTH-trained checkpoints incorrectly translate the surname "Gray" as "Šedivý". To gain mathematical insight into this phenomenon, we computed the relative probability of the translation preferred by SYNTH and AUTH-block trained networks (B). The graph shows the relative probability of using the translation "chlap" (blue) vs. "člověk" and the relative probability of "Gray" (green) vs. "Šedivý" by block-BT models with (bold line) and without (dashed line) averaging. It shows that the model without averaging prefers "chlap" in SYNTH-trained checkpoints (white background) and "člověk" in AUTH-trained checkpoints (purple background), but the SYNTH-trained checkpoints show a much greater confidence than the AUTH-trained ones. Therefore, the translation "chlap" is preferred by the model with averaging not only in the SYNTH-trained checkpoints, but also in multiple following checkpoints, as the higher probability enables selection of lower-frequency translation. This example suggests that the increased diversity of networks and translations via distinct SYNTH and AUTH blocks of training data reflects the fact that the corresponding networks specialize in different aspects of translation. At the same time, they implicitly keep track of their confidence in each given translation, which is taken into account by checkpoint averaging. This is also what may lead to a translation which is relatively acceptable to all averaged networks, while not being preferred by any as the first choice (Fig. 7B). We compute the relative probability as follows: We score both translation variants using the `t2t-decoder --score_file` command, which gives us a negative log-likelihood (corresponding to the training loss) of the translation assigned by a given model checkpoint. Relative probability of translation A vs. translation B is then computed as exp(-scoreA) / (exp(-scoreA) + exp(-scoreB)). In these experiments, translations A and B differ only in a single word (e.g. "chlap" vs. "člověk") and the same source sentence is provided for the scoring.

3. Supplementary Tables

data set	sentence pairs (k)	EN words (k)	CS words (k)
WMT2018 authentic train	58 085	641 942	563 168
– CzEng 1.7	57 065	618 424	543 184
– Europarl v7	647	15 625	13 000
– News Commentary v12	211	4 544	4 057
– CommonCrawl	162	3 349	2 927
EN NewsCrawl 2016–2017	47 483	934 981	-
CS NewsCrawl 2007–2017	65 383	-	927 348
total	170 951	1 576 923	1 490 516

Supplementary Table 1. English-Czech training data sizes (in thousands). In the case of monolingual data (EN and CS NewsCrawl), the first column reports the number of sentences; in the case of parallel data it reports the number of sentence pairs.

data set	sentence pairs (k)	EN words (k)	CS words (k)
WMT13	3.0	56	48
– WMT13-orig-en	2.5	46	40
– WMT13-orig-cs	0.5	10	8
WMT17	3.0	55	47
WMT18	3.0	56	47
– WMT18-orig-en	1.5	31	19

Supplementary Table 2. English-Czech development and test data sizes (in thousands).

data set	sentence pairs (k)	EN words (k)	FR words (k)
WMT2014 authentic train	34 335	911 755	1 044 000
EN NewsCrawl 2011–2017	127 554	2 655 883	
FR NewsCrawl 2008–2014	37 320		780 353
Total	199 209	3 567 638	1 824 353

Supplementary Table 3. English-French training data sizes (in thousands).

data set	sentence pairs (k)	EN words (k)	FR words (k)
WMT14	3	62	70
WMT12+WMT13	6	120	131

Supplementary Table 4. English-French development and test data sizes (in thousands).

data set	sentence pairs (k)	EN words (k)	PL words (k)
WMT2020 authentic train	10 789	210 481	185 865
EN NewsCrawl 2018	22 691	422 603	
PL NewsCrawl 2018–2019	3 787		56 570
total	37 267	633 084	242 435

Supplementary Table 5. English-Polish training data sizes (in thousands).

data set	sentence pairs (k)	EN words (k)	PL words (k)
newsdev2020	4	41	36

Supplementary Table 6. English-Polish development data sizes (in thousands).

	P(A)	P(E)	Kappa
**Adequacy**	0.533	0.347	0.285
**Fluency**	0.504	0.345	0.244
**Overall**	0.511	0.348	0.250

Supplementary Table 7. IAA Kappa results for sentence-level context-aware evaluation of CUBBITT vs. Reference. P(A) is the raw agreement, i.e. the ratio of comparisons where a pair of evaluators agreed on the ranking (better, worse, tie). P(E) is the expected agreement by chance, estimated using a single marginal distribution for all evaluators. Kappa = (P(A) – P(E)) / (1 – P(E)).

	P(A)	P(E)	Kappa
**Addition**	0.788	0.615	0.449
**Omission**	0.669	0.615	0.141
**Ambiguous**	0.703	0.679	0.075
**Shift**	0.669	0.578	0.217
**Other adequacy**	0.585	0.568	0.039
**Grammar**	0.763	0.756	0.026
**Spelling**	0.949	0.918	0.380
**Other fluency**	0.390	0.384	0.010
**Context**	0.797	0.743	0.209

Supplementary Table 8. IAA Kappa results for error-type context-aware evaluation of CUBBITT vs. Reference.

	P(A)	P(E)	Kappa
**Adequacy**	0.617	0.333	0.426
**Fluency**	0.537	0.335	0.304

Supplementary Table 9. IAA Kappa results for sentence-level context-aware evaluation of five MT systems.

	P(A)	P(E)	Kappa
**Cubbitt vs. Human**	0.601	0.504	0.195
**Google vs. Human**	0.688	0.526	0.342
**All**	0.624	0.513	0.228

Supplementary Table 10. IAA Kappa results for the Translation Turing test.

4. Supplementary References

1. Bojar, O. *et al.* The Joy of Parallelism with CzEng 1.0. in *Proceedings of the Eighth International Language Resources and Evaluation Conference (LREC'12)* 3921–3928 (2012).

2. Sennrich, R., Haddow, B. & Birch, A. Edinburgh Neural Machine Translation Systems for WMT 16. in *Proceedings of the First Conference on Machine Translation (WMT)* 371–376 (2016).

3. Bojar, O. *et al.* Findings of the 2016 Conference on Machine Translation. in *Proceedings of the First Conference on Machine Translation: Volume 2, Shared Task Papers* 131–198 (2016). doi:10.18653/v1/W16-2301
