# Supplementary material for: Transforming machine translation: a deep learning system reaches news translation quality comparable to human professionals
Source: Nat Commun. 2020 Sep 1;11:4381. doi: 10.1038/s41467-020-18073-9 (PMC7463233; doi:10.1038/s41467-020-18073-9)
Supplement: Supplementary file 3 — Description of Additional Supplementary Files [file 41467_2020_18073_MOESM3_ESM.pdf]

Description of Additional Supplementary Files

File Name: Supplementary Data 1
Description: Examples of translation errors and properties of the reference human translation and CUBBITT. The data extend Figure 5, providing Czech translations by the human reference and CUBBITT, as well as the values of the manual evaluation for the individual sentences.
