# Supplementary material for: Transforming machine translation: a deep learning system reaches news translation quality comparable to human professionals
Source: Nat Commun. 2020 Sep 1;11:4381. doi: 10.1038/s41467-020-18073-9 (PMC7463233; doi:10.1038/s41467-020-18073-9)
Supplement: Supplementary file 5 — Reporting Summary [file 41467_2020_18073_MOESM5_ESM.pdf]

nature research

Corresponding author(s): *Double-blind peer review submissions: write DBPR and your manuscript number here instead of author names.*

Last updated by author(s): *YYYY-MM-DD*

Reporting Summary

Nature Research wishes to improve the reproducibility of the work that we publish. This form provides structure for consistency and transparency in reporting. For further information on Nature Research policies, see our Editorial Policies and the Editorial Policy Checklist.

Statistics

For all statistical analyses, confirm that the following items are present in the figure legend, table legend, main text, or Methods section.

n/a	Confirmed	
☐	☒	The exact sample size (n) for each experimental group/condition, given as a discrete number and unit of measurement
☐	☒	A statement on whether measurements were taken from distinct samples or whether the same sample was measured repeatedly
☐	☒	The statistical test(s) used AND whether they are one- or two-sided<br>*Only common tests should be described solely by name; describe more complex techniques in the Methods section.*
☐	☒	A description of all covariates tested
☐	☒	A description of any assumptions or corrections, such as tests of normality and adjustment for multiple comparisons
☐	☒	A full description of the statistical parameters including central tendency (e.g. means) or other basic estimates (e.g. regression coefficient) AND variation (e.g. standard deviation) or associated estimates of uncertainty (e.g. confidence intervals)
☐	☒	For null hypothesis testing, the test statistic (e.g. F, t, r) with confidence intervals, effect sizes, degrees of freedom and P value noted<br>*Give P values as exact values whenever suitable.*
☒	☐	For Bayesian analysis, information on the choice of priors and Markov chain Monte Carlo settings
☐	☒	For hierarchical and complex designs, identification of the appropriate level for tests and full reporting of outcomes
☐	☒	Estimates of effect sizes (e.g. Cohen's d, Pearson's r), indicating how they were calculated

Our web collection on statistics for biologists contains articles on many of the points above.

Software and code

Policy information about availability of computer code

Data collection	The neural networks were training using the Tensorflow library. Langid toolkit was used for detection of languages (Methods 4). Spreadsheets for annotators were created and provided using Google Sheets.
Data analysis	Data analyses were carried out using Matlab (2017b).

For manuscripts utilizing custom algorithms or software that are central to the research but not yet described in published literature, software must be made available to editors and reviewers. We strongly encourage code deposition in a community repository (e.g. GitHub). See the Nature Research guidelines for submitting code & software for further information.

Data

Policy information about availability of data

All manuscripts must include a data availability statement. This statement should provide the following information, where applicable:

- Accession codes, unique identifiers, or web links for publicly available datasets
- A list of figures that have associated raw data
- A description of any restrictions on data availability

Data used for comparison of human and machine translations may be downloaded at http://hdl.handle.net/11234/1-3209.

Field-specific reporting

Please select the one below that is the best fit for your research. If you are not sure, read the appropriate sections before making your selection.

- [ ] Life sciences
- [x] Behavioural & social sciences
- [ ] Ecological, evolutionary & environmental sciences

For a reference copy of the document with all sections, see nature.com/documents/nr-reporting-summary-flat.pdf

Behavioural & social sciences study design

All studies must disclose on these points even when the disclosure is negative.

Study description	Human evaluators were asked to quantitatively assess quality of presented translations.
Research sample	The evaluated translated sentences were sampled from WMT18 data set. In each of the available 52 documents, ten consecutive sentences were randomly chosen and scored by human evaluators. Three groups of paid evaluators were recruited: 6 professional translators, 3 translation theoreticians and 7 other evaluators (non-professionals). All 16 evaluators were native Czech speakers with excellent knowledge of the English language. The professional translators were required to have at least eight years of professional translation experience and they were contacted via The Union of Interpreters and Translators (http://www.jtpunion.org/). The translation theoreticians were from The Institute of Translation Studies, Charles University's Faculty of Arts (https://utrl.ff.cuni.cz/). In total, six of the 16 evaluators were male. Each evaluator scored 9 non-spam documents, with up to 10 sentences in each document. Every document (and thus also every sentence) was scored by at least two evaluators, whose scores were avaraged in the main analysis. In total, 512 unique sentences were evaluated (in the main analysis) by the 15 evaluators who passed quality control.
Sampling strategy	The documents were assigned to evaluators randomly in such a way that every document was scored by at least two different evaluators. The sample-size (number of evaluated unique sentences) was determined with power calculation for sign-test (using matlab sampsizepwr function) to be at least 187 sentences, such that difference of 10% between quality of the two evaluated systems can be distinguished with power of 0.8.
Data collection	Each evaluator was sent a link to a different Google sheet table with translated sentences, where the order of evaluated systems was blinded (and randomised over sections of rows, which represented translated documents). Both the preparation of the documents (including the randomisation) and evaluation were performed by a custom matlab script (available to download).
Timing	The start of data collection was 22 August 2018 and the end was 9 December 2018.
Data exclusions	Quality control and inclusion/exclusion criteria were preestablished using "spam document" (please see Supplementary Materials section 5.3 quality control). One evaluator did not pass the quality control and was therefore excluded from the analysis.
Non-participation	No evaluators dropped out of the study after confirming to participate.
Randomization	Allocation of documents to evaluators was random, performed by a custom matlab script (available for download), as well as randomisation of orders of the evaluated systems within documents.

Reporting for specific materials, systems and methods

We require information from authors about some types of materials, experimental systems and methods used in many studies. Here, indicate whether each material, system or method listed is relevant to your study. If you are not sure if a list item applies to your research, read the appropriate section before selecting a response.

Materials & experimental systems

n/a	Involved in the study
[x]	[ ] Antibodies
[x]	[ ] Eukaryotic cell lines
[x]	[ ] Palaeontology and archaeology
[x]	[ ] Animals and other organisms
[x]	[ ] Human research participants
[x]	[ ] Clinical data
[x]	[ ] Dual use research of concern

Methods

n/a	Involved in the study
[x]	[ ] ChIP-seq
[x]	[ ] Flow cytometry
[x]	[ ] MRI-based neuroimaging
