# Supplementary material for: Transforming machine translation: a deep learning system reaches news translation quality comparable to human professionals
Source: Nat Commun. 2020 Sep 1;11:4381. doi: 10.1038/s41467-020-18073-9 (PMC7463233; doi:10.1038/s41467-020-18073-9)
Supplement: Supplementary file 2 — Peer Review File [file 41467_2020_18073_MOESM2_ESM.pdf]

Reviewers' comments:

Reviewer #1 (Remarks to the Author):

The authors present research where they detail a heavily engineered machine translation (MT) system (called CUBBITT) for the English-Czech language pair which returns state-of-the-art automatic metric scores. They go on to show that this system's output is rated higher for accuracy than the human reference translations which were produced by a translation agency.

There have been a number of papers already claiming that their machine translation output is reaching parity with human quality (Wu et al 2016, Hassan et al 2018). These results were investigated further and it was seen that for sentences in isolation, MT systems can perform better than humans but that MT systems do worse when sentences are evaluate in context of their document (Laubli et al 2018). The paper under discussion shows careful human evaluation of sentences in their document context, and it is the first research to show that their MT systems do better than the human reference when evaluated in this way.

I think that this paper presents an interesting and very thorough human analysis of the strengths of the MT output. They compare professional translators with non-professional translators. They separate out accuracy and fluency, and they show that MT is more accurate and less fluent than humans.

The most interesting results are where the authors try to tease apart how exactly the MT systems outperform humans by counting sentences with different error types. Their CUBBITT model does noticeably better on omissions, insertions and shifts. This leads me to wonder what was happing with the human translations. Translators are often under time pressures and can be somewhat loose with their translations. MT systems are being rewarded for being more literal and getting slightly lower fluency scores as a result.

I would be interested to know how important the human omissions/insertions might be, perhaps they are not core to meaning of the sentences. Humans crucially bring an understanding of salience to the problem. What are the most important facts to convey? If there are 10 facts, perhaps only the first 9 are relevant and important to mention. Humans will also correctly relate people and facts to each other. I would like to know if the "other fluency" category includes errors where perhaps the MT is more prone to mistakes like making subjects into objects, or attaching clauses to the wrong head. These would cause significant mistakes in the meaning of the translation, not just in the fluency.

I still believe that conscientious human translators can be trusted to convey the meaning of the source text better than MT systems. I consider that the domain being tested is quite a general domain with sentences of average or below average length and that the findings would likely not hold for more difficult texts.

In summary, this paper is a strong contribution to the field of Computational Linguisitics. However I do not think that this is a paper that has findings which are so important and novel and interesting to a wider audience that they are worthy of dissemination in Nature.

Strengths:

- The major contribution of the paper is to propose a number of different methods for evaluating human parity with machine translation
- It is also novel that they show that their model beats humans references in rigorous in document level evaluations.
- The idea of block-backtranslation, where the training alternates large blocks of true parallel and

synthetic data being better than mixing them both in training is interesting and possibly relevant to other MT research groups. However, it would have been nice for authors to suggest an explanation for this working so well.

Weakness:

- Their model has only been assessed for one translation direction which has a lot of high quality data on straightforward news translation data. This does not test the limits of their model and their approach. CUBBITT is heavily engineered and could be very brittle. I could easily imagine that CUBBITT would fail to beat human translations in another language pair, with a harder test set, or with slightly less high quality training data. These might just be the only circumstances under which CUBBITT can actually beat humans. This is of course still impressive, but it does not help other researchers to assess the general relevance of the final result, or of their particular model/training regime.
- They didn't look into the quality of the human reference translations. I think this is key to the debate - poor human translations will be straightforward to improve upon.
- Of the sentences used in the human evaluation results, were they all original English sentences? Or were they mixed with Original Czech translated into English. This is not made clear in the paper and could be a confounding factor as the English source could be poor quality or even just simpler and then easier to translate with MT. (This is mentioned in Lines 157-160 but it is not clarified)

More minor questions:
- Line 134: This optimal ratio of 6:2 for mixing models trained with authentic and synthetic leads to the peaks in BLEU. However

- Figure 3A does not add much to the paper and none of the other systems in this figure are described in meaningful detail or are indeed relevant to the paper

- Is 2A the training run for the final MT4 model shown in 2B? This means that the model

- Was the translation Turing test 264 document level? If not then the conclusions would be weaker

- References missing for many titles (8, 9, 10, 17, 20, 21)

Reviewer #2 (Remarks to the Author):

The main contributions of this paper are Iterative block back-translation and a very detailed human evaluation of machine translation output vs human-created reference translations for English-Czech WMT18 test sets. Especially the human evaluation results presented in this paper deserve recognition, it is rare to see such a detailed analysis with that many different angles (will the results be made public?).

The iterative block backtranslation technique seems to show that a system that is trained on piecewise alternating segments for authentic and synthetic translations (back-translation) seems to results in a kind of ladder-climbing effect when combined with checkpoint averaging where the new maximum after each such alternated phase stays well above the maxima of the actual unaveraged parameters trained. These effects seem to improve even more when repeated in an iterative fashion where the gradually improved models provide the synthetic translations for the next iteration of models. This method combined with other good practices for training modern NMT systems result in state-of-the-art systems for English-Czech and Check-English translation at WMT18.

This work further analyzes the outputs of these systems in a detailed human evaluation. Different from WMT18, a source-based analysis that differs significantly from the accepted WMT18 evaluation scheme but using source-based analysis (allows to judge reference quality), adding context-aware judgements, and splitting into adequacy and fluency. The authors also add a “translation Turing task” where professionals and non-professionals are tasked to tell apart human translation from machine translation. This is all very commendable, and I would like to see more papers like that. They are too rare. The authors make a strong case that their system has indeed reached human or even super-human translation quality, the extensive analysis support this. The quality of the analysis is very high, the graphical presentation is clear and visually pleasing.
This is a very well written and presented paper and I would recommend it without much revision for publication if the current date was February 2019. But I am reviewing this work end of February 2020 and cannot ignore that the whole previous year happened. The biggest problem I have with the paper is the lack of integration of current results from after WMT 2018. WMT 2019 took place in April 2019; results were published in the summer of 2019; and a lot of the results presented in this paper have been seen in similar form for other languages with similar context-based evaluation methods (http://www.statmt.org/wmt19/pdf/53/WMT01.pdf).

I am guessing that this work has been written up before that and might have been under review for a long time, but the results of WMT19 have been public since summer 2019. Especially in the light of the strongly worded claims of potentially revolutionary results, it is a bit unfortunate that this revolution might have happened elsewhere while this paper was potentially held back for publication/review reasons.

The results from WMT 2019 for English-Czech specifically, also undermine the claims from the paper. The newest system for the English-Czech translation task (CUNI-Transformer) that followed relatively similar evaluation guidelines to the ones proposed in this paper did not beat the human reference in terms of general translation quality; the human reference has been judged to be significantly better. Of course, we are missing here the split into adequacy and fluency that the authors propose, but a comment from the authors on that part would be direly needed as it questions the repeatability of their results for newer or other test sets even in the news domain, not to mention other domains.
Lines 310-313 are such a strongly worded claim which is problematic the moment the authors step out of the news domain for which these systems were specifically built. I agree that human translators are not necessarily the upper bound for translation quality, but that has been shown only for one language pair, for one test set in one domain. Generalizing from that alone seems overconfident. The WMT2019 results across multiple languages, especially en-de and de-en, might give that more credence as a full picture, but for that they would have to be at least mentioned in this paper.

The other big question which I find has not been answered to my full satisfaction is how has the feat of superhuman quality actually been achieved? I would have liked to see a verification of the iterative block back-translation method for other languages than just translations between Czech and English, even if done using automatic metrics. It seems this is the only meaningful improvement presented in this paper over other transformer-based baselines, so it would be solely responsible for achieving superhuman translation quality (or for at least getting over that threshold)? Is that repeatable for other languages, for other domains? Is the Czech-English parallel training corpus somehow special? These things at least require more comments. The authors hint at the possibility that this is a compounding effect (314-322) of multiple factors but do so in the conclusions only.

Smaller comments or questions (in no particular order):
• In the references I recommend replacing the Arxiv links with ACL Anthology links wherever possible. Many of the cited works have been published at conferences in parallel to Arxiv versions and these should be considered canonical.

• What role might test set quality variability play considering the lack of superhuman results for English-Czech in WMT2019?
• How does current evaluation practice of WMT2019 differ from the one presented?
• Please provide a date for when your translations have been collected from Google Translate etc. They are likely to change over time and should be marked with something like a timestamp.
• Lines 153-154 need to makes clear that this is the case for the language pairs investigated, otherwise it sounds hyperbolic, since you we don't know what was going on for the other language pairs.
• The paper is well written and presented although the limitations of the journal seem to have resulted in moving many important parts into the supplementary material. This seems to make the paper a bit incomplete without them: one such example is the so-called "translationese tuning" which is not clear without consulting the supplementary. This is a minor point.

My final comment would be: this is a good paper, but as it stands now, it has been overtaken by the developments of 2019 and needs updating before publication. The results need to be put into a larger context, including specifically results from WMT2019.

Dear reviewers,

Thank you for your valuable comments. Please find below how we addressed the individual issues.

Reviewer #1 (Remarks to the Author):

The authors present research where they detail a heavily engineered machine translation (MT) system (called CUBBITT) for the English-Czech language pair which returns state-of-the-art automatic metric scores. They go on to show that this system's output is rated higher for accuracy than the human reference translations which were produced by a translation agency.

There have been a number of papers already claiming that their machine translation output is reaching parity with human quality (Wu et al 2016, Hassan et al 2018). These results were investigated further and it was seen that for sentences in isolation, MT systems can perform better than humans but that MT systems do worse when sentences are evaluate in context of their document (Laubli et al 2018). The paper under discussion shows careful human evaluation of sentences in their document context, and it is the first research to show that their MT systems do better than the human reference when evaluated in this way.

I think that this paper presents an interesting and very thorough human analysis of the strengths of the MT output. They compare professional translators with non-professional translators. They separate out accuracy and fluency, and they show that MT is more accurate and less fluent than humans.
Thank you for your useful comments and suggestions. Please see below how we addressed them individually.

The most interesting results are where the authors try to tease apart how exactly the MT systems outperform humans by counting sentences with different error types. Their CUBBITT model does noticeably better on omissions, insertions and shifts. This leads me to wonder what was happing with the human translations. Translators are often under time pressures and can be somewhat loose with their translations. MT systems are being rewarded for being more literal and getting slightly lower fluency scores as a result.
We agree that time pressure is a likely factor contributing to the quality of translations by a translation agency compared to an MT system. That said, this is a part of the reality of translation by professional agencies. We highlighted in the Discussion that a carefully conducted translation by an expert with a large amount of time was not our benchmark and that it may be unreachable by current MT systems.

We also agree that more literal translations can lead to lower fluency scores (in fact, from the optional text comments in the evaluation, we know that this did happen at least sometimes). At the same time, CUBBITT's fluency is often excellent, and it is capable of nonliteral sentence restructuring to fit Czech writing style better: we added an example of nontrivial sentence restructuring in Fig. 5F. Further evidence showing that CUBBITT is capable of translation that is not obviously machine-like is the translation Turing test.

I would be interested to know how important the human omissions/insertions might be, perhaps they are not core to meaning of the sentences. Humans crucially bring an understanding of salience to the problem. What are the most important facts to convey? If there are 10 facts, perhaps only the first 9 are relevant and important to mention.
We have added several translation examples to illustrate the common problems in the human and CUBBITT translations in Fig. 5. The human omission/insertion/shift of meaning types of errors frequently contained an error which is easy to make for a human translator (especially under time pressure) and/or is unintentional, but can lead to a major change of the meaning of the sentence.

For instance, the word Scottish is used twice in the following sentence and on the first sight the first occurrence might have seemed redundant to the human translator, however when omitted (by the human reference translation), the meaning of the sentence is changed considerably (a player not playing in Scotland since 2013 versus the player not playing at all since 2013).
"This event will be his first Scottish appearance since the Aberdeen Asset Management Scottish Open in 2013."

In a different example, the human translator accidentally read *"Democratics and conservation groups"* **as** *"Democratics and conservative groups"***, which is an error that one can easily imagine happening to a human translator (especially under time pressure, and with the background knowledge that a political article is being translated), but which markedly changes the meaning of the sentence.**

"But the efforts have triggered pushback by Democratics and conservation groups who are concerned about the impact of greater emissions on public health."

Obviously not all omission/insertion/shift of meaning types of errors caused a major change of the meaning of the sentence. For instance, in the last translation example, the human translator also omitted the word "greater", causing only a slight change of meaning of the second half of the sentence. However, our evaluators were asked to assign the adequacy score according to "*the degree to which the meaning of the source sentence is preserved in the translation*", allowing the less important errors to be evaluated by a smaller decrease of the adequacy score. Moreover, our evaluation was document-aware, so if there was a fact mentioned earlier (or later) in the document and omitted from the

current sentence, the evaluators were not instructed to annotate it as an adequacy error, nor to decrease the adequacy/overall quality scores.

Humans will also correctly relate people and facts to each other. I would like to know if the "other fluency" category includes errors where perhaps the MT is more prone to mistakes like making subjects into objects, or attaching clauses to the wrong head.

As we have not defined this as one of the types of errors, we unfortunately cannot quantify its occurrence formally. However, from our experience and from the optional comments in the evaluation, CUBBITT does not tend to make such mistakes. In fact, it shows a surprising ability to learn the relationships between subjects and objects in the sentence even in long and complicated sentences. This can be seen in the above-mentioned example in Fig. 5F, where the order of individual clauses is markedly changed in order to obtain better fluency without any change in sentence meaning.

Based on the optional comments in the evaluation, the “other fluency” category was often used in cases when CUBBITT’s translations were too literal, while the human reference loosely translated the meaning of the sentence using a completely different wording (and broader knowledge of the subject).

These would cause significant mistakes in the meaning of the translation, not just in the fluency.
We completely agree, in such a case, the evaluators should decrease both the fluency and the adequacy scores (and of course also the overall quality).

I still believe that conscientious human translators can be trusted to convey the meaning of the source text better than MT systems.
We agree that highly qualified and experienced human translators with sufficient time and resources will likely produce better translations than any MT system. However, many clients will not have the resources to find and pay such translators, highly qualified in the relevant domain. We believe MT systems can be extremely helpful for this need, providing extremely fast translations of high quality, approaching (or even surpassing, in certain aspects) less expensive (but still vastly more expensive than a machine translation) human translators. We have updated the discussion and conclusions of the manuscript to make this message clear.

I consider that the domain being tested is quite a general domain with sentences of average or below average length and that the findings would likely not hold for more difficult texts.
We have extended the results with an analysis of the effect of sentence length on the evaluated quality of CUBBITT compared to the human reference. Interestingly, CUBBITT compared to the reference performs better in the longer than the shorter sentences with regards to adequacy, fluency, and overall quality (Fig. S8). This result may not be so

surprising as it might initially look: longer sentences may give more material for the application of deep learning attention, which enables correct gender assignment, provides more context to guess the correct translation of a word with multiple meanings, etc.

Inspired by your comment, we computed the average English sentence length in our context-aware evaluation; it is 21.8 words (excluding punctuation) and the median is 21. The average sentence length in two popular English resources we checked are:
- 17.6 words in the written part of British National Corpus (http://www.natcorp.ox.ac.uk/docs/URG/BNCdes.html#BNCcompo, 10 words in the spoken part) and
- 15.3 in the English Web Treebank (https://catalog.ldc.upenn.edu/LDC2012T13).
- Elements of Style for Writing Scientific Journal Articles reports that the "average length of sentences in scientific writing is only about 12-17 words" (https://www.gfdl.noaa.gov/wp-content/uploads/2018/08/Elements_of_Style.pdf).
Overall, we found no evidence suggesting that the sentences in our dataset were overly short, rather on the contrary.

Nevertheless, we agree that our results at this point cannot be generalised beyond the evaluated domain of the news articles before any further evaluations of more general domains (and possibly also additional training on domain-specific data sets) are performed. We have made this clear in the discussion of the manuscript.

In summary, this paper is a strong contribution to the field of Computational Linguisitics. However I do not think that this is a paper that has findings which are so important and novel and interesting to a wider audience that they are worthy of dissemination in Nature.
Automated language translation is such a widely used application, with vast amounts of users in the general public, as well as news agencies, students, scientists, etc., that we believe the wide readership of the Nature Communications journal will be interested to read about an important milestone of the field, which is a machine translation getting so close to the quality of human translation that it can even surpass it in certain aspects.

Detection of this milestone is the main contribution of our study. Importantly, our evaluation methodology was very thorough and considerably fairer than previous approaches, such as by considering the document context. It also brings much more insight, e.g., separating fluency and adequacy, and annotating types of translation errors. Given the advantages of this methodology, we believe our results are trustworthy and the statement that the milestone is reached is credible.

The main technological novelty of our study is the iterated block-backtranslation combined with checkpoint averaging, which was used for the training of our CUBBITT system and which our evaluations suggest to be crucial for the success of the system.

Based on your suggestion (in "Strengths" below), we have now added a sequence of analyses which provide insight into why and how the method improves the translations.

Strengths:
- The major contribution of the paper is to propose a number of different methods for evaluating human parity with machine translation
- It is also novel that they show that their model beats humans references in rigorous in document level evaluations.
- The idea of block-backtranslation, where the training alternates large blocks of true parallel and synthetic data being better than mixing them both in training is interesting and possibly relevant to other MT research groups. However, it would have been nice for authors to suggest an explanation for this working so well.

Thank you for appreciating the strengths of this work. Based on the feedback of both reviewers, we conducted additional investigation into the mechanisms underlying the improved translation performance by the block backtranslation combined with checkpoint averaging (please see the new section "Generality of block backtranslation and why does it improve translation?"). The results show that the alternation of authentic and synthetic data in block-BT leads to increased diversity of the translations (Fig. S16), which is leveraged by checkpoint averaging to generate novel translations, not used in the past by the model without averaging (Fig. 8A). We show that these novel translations largely contribute to the increased performance as measured by BLEU (Fig. 8B). Finally, we show how this synergy can work in concrete sentence examples. For instance, in Fig. 7A and S17, the translation produced by block-BT with checkpoint averaging contains two phrases which were correctly translated only by the models trained in the authentic blocks, while another phrase was correctly translated only by the models trained in the synthetic blocks. Only after the checkpoint averaging of both types of models, the system combined all three phrases correctly to form a good translation.

Weakness:
- Their model has only been assessed for one translation direction which has a lot of high quality data on straightforward news translation data. This does not test the limits of their model and their approach. CUBBITT is heavily engineered and could be very brittle. I could easily imagine that CUBBITT would fail to beat human translations in another language pair, with a harder test set, or with slightly less high quality training data. These might just be the only circumstances under which CUBBITT can actually beat humans. This is of course still impressive, but it does not help other researchers to assess the general relevance of the final result, or of their particular model/training regime.

We agree that the generality of CUBBITT's success to other language pairs remains to be evaluated and we have highlighted this limitation in the discussion. However, we would like to note that CUBBITT has not been as heavily engineered as it may seem. The Transformer architecture itself has been successfully used before for a number of language pairs (Bojar et al., 2018). The only Czech-specific component of CUBBITT is the

Regex postediting (mainly of Czech quotation symbols, see Supplementary Materials 3.6). However, our manual evaluation showed that the main improvement gained was through block-BT with checkpoint averaging, while the combination of iteration of block-BT, translationese tuning, and regex postediting brought only a minor and non-significant improvement in adequacy and fluency (Fig. 4B).

In order to validate the benefits of block-BT with checkpoint averaging on a different language pair at least using automatic metrics, as suggested by the second reviewer, we have now trained CUBBITT also for the English-French and English-Polish language pairs (in both directions). Although we have not performed any language-specific tuning, nor any other engineering, we have observed similar results as obtained on the English-Czech pair. In particular, the new language pairs also showed a synergy between block-BT and checkpoint averaging and the combined model in its peaks clearly outperformed mix-BT in all the four new language translation directions (Fig. S14).

Finally, our newly added analyses of the synergy between block-BT and checkpoint averaging bring insights into how and why it works, suggesting it to be a general language-independent principle (albeit dependent on the existence of both parallel and monolingual training data).

- They didn't look into the quality of the human reference translations. I think this is key to the debate - poor human translations will be straightforward to improve upon.
We agree that the quality of the human reference is an important factor.

We note that WMT18 Proceedings (Bojar et al., 2018) describe the WMT18 reference translations as follows:
In particular, the Czech and German test sets were translated to/from English by the professional level of service of Translated.net, preserving 1-1 segment translation and aiming for literal translation where possible. Each language combination included 2 different translators: the first translator took care of the translation, the second translator was asked to evaluate a representative part of the work to give a score to the first translator. All translators translate towards their mother tongue only and need to provide a proof or their education or professional experience, or to take a test; they are continuously evaluated to understand how they perform on the long term. The domain knowledge of the translators is ensured by matching translators and the documents using TRank, http://www.translated.net/en/T-Rank.

As mentioned above, we have added a new paragraph into the Discussion on the quality of the human reference translations.

- Of the sentences used in the human evaluation results, were they all original English sentences? Or were they mixed with Original Czech translated into English. This is not made

clear in the paper and could be a confounding factor as the English source could be poor quality or even just simpler and then easier to translate with MT. (This is mentioned in Lines 157-160 but it is not clarified)

Yes, all our human evaluations were performed on originally English sentences only. This information was included in the sections 2.2 and 4 in the Supplementary Materials, but we have now clarified this also in the main text of the manuscript.

More minor questions:

- Line 134: This optimal ratio of 6:2 for mixing models trained with authentic and synthetic leads to the peaks in BLEU. However

Unfortunately, given the second sentence is mostly missing, we cannot answer this.

- Figure 3A does not add much to the paper and none of the other systems in this figure are described in meaningful detail or are indeed relevant to the paper

Figure 3A shows that top academia and commercial systems struggle to significantly outperform the human reference (which among other things means the quality of the reference was not poor and easy to surpass). Although the figure is a reanalysis of data previously published in the findings of WMT18 results (Bojar et al., 2018), we provide the statistics and visualisation identical with the rest of our manuscript, aiding easier comparison with the following results. Finally, we also reanalysed the data, confirming that the results hold also on the original English sentences only (Fig. S4).

The three commercial systems are Yandex (online G), Microsoft/Bing Translator (online A), and Google Translate (online B). We could not reveal the identity of them publicly, as the rules of the WMT do not allow that.

- Is 2A the training run for the final MT4 model shown in 2B? This means that the model

No, the block-BT+avg8 training run shown in Fig. 2A is the first iteration of block-backtranslation and therefore corresponds to MT2 in the general diagram in Fig. 2B and in the detailed diagram in Fig. S2. We have clarified this in the legend of Fig. 2.
The final MT4 model has a BLEU curve (not shown) with a similar shape as MT2, but about 0.5 BLEU higher.

- Was the translation Turing test 264 document level? If not then the conclusions would be weaker

The Turing test was on the sentence level. We made this decision after careful consideration of our resources for the test and other factors, such as that the Turing test was only a secondary result in our study. If we performed the Turing test on the document level, the participants would know that all the ca. 10 sentences in the document come from the same source. We would therefore need to increase the number of evaluated sentences ca. 10 times in order to keep the same statistical power, which was beyond our resources. While we agree that the results would be even stronger on a document level,

we nevertheless thought the sentence-level results to be a very interesting secondary result. We have clarified in the main text of the manuscript that the Turing test was sentence-level:
We therefore conducted a <u>sentence-level</u> "Translation Turing test", in which participants were asked to judge whether a translation of a sentence was performed by a machine or a human on 100 independent sentences (the source sentence and a single translation was shown).

- References missing for many titles (8, 9, 10, 17, 20, 21)
Thank you, we have updated the references and their titles.

Reviewer #2 (Remarks to the Author):

Thank you for your useful comments and suggestions. Please see below how we addressed them individually.

The main contributions of this paper are Iterative block back-translation and a very detailed human evaluation of machine translation output vs human-created reference translations for English-Czech WMT18 test sets. Especially the human evaluation results presented in this paper deserve recognition, it is rare to see such a detailed analysis with that many different angles (will the results be made public?).
Yes, all the English→Czech evaluation data, scripts, and results are made publicly available through http://hdl.handle.net/11234/1-3209.

The iterative block backtranslation technique seems to show that a system that is trained on piecewise alternating segments for authentic and synthetic translations (back-translation) seems to results in a kind of ladder-climbing effect when combined with checkpoint averaging where the new maximum after each such alternated phase stays well above the maxima of the actual unaveraged parameters trained. These effects seem to improve even more when repeated in an iterative fashion where the gradually improved models provide the synthetic translations for the next iteration of models. This method combined with other
good practices for training modern NMT systems result in state-of-the-art systems for English-Czech and Check-English translation at WMT18.

This work further analyzes the outputs of these systems in a detailed human evaluation. Different from WMT18, a source-based analysis that differs significantly from the accepted WMT18 evaluation scheme but using source-based analysis (allows to judge reference quality), adding context-aware judgements, and splitting into adequacy and fluency. The authors also add a "translation Turing task"; where professionals and non-professionals are tasked to tell apart human translation from machine translation. This is all very

commendable, and I would like to see more papers like that. They are too rare. The authors make a strong case that their system has indeed reached human or even super-human translation quality, the extensive analysis support this. The quality of the analysis is very high, the graphical presentation is clear and visually pleasing.
We thank you for appreciating the strengths of our work.

This is a very well written and presented paper and I would recommend it without much revision for publication if the current date was February 2019. But I am reviewing this work end of February 2020 and cannot ignore that the whole previous year happened. The biggest problem I have with the paper is the lack of integration of current results from after WMT 2018. WMT 2019 took place in April 2019; results were published in the summer of 2019; and a lot of the results presented in this paper have been seen in similar form for other languages with similar context-based evaluation methods (http://www.statmt.org/wmt19/pdf/53/WMT01.pdf). I am guessing that this work has been written up before that and might have been under review for a long time, but the results of WMT19 have been public since summer 2019. Especially in the light of the strongly worded claims of potentially revolutionary results, it is a bit unfortunate that this revolution might have happened elsewhere while this paper was potentially held back for publication/review reasons.
We completely agree with you that the field has made a huge leap in the year 2019. We worked hard to finish our evaluation of the WMT18 results presented in this manuscript and publish it as soon as possible. We managed to do so and submit the manuscript in December 2018, since when it has been in review (in Science and then Nature Communications).

However, we agree with you that the WMT19 results need to be commented on in our manuscript, and we have adjusted our discussion accordingly. We have also removed the "*potentially revolutionary*" claim that you mentioned.

The results from WMT 2019 for English-Czech specifically, also undermine the claims from the paper. The newest system for the English-Czech translation task (CUNI-Transformer) that followed relatively similar evaluation guidelines to the ones proposed in this paper did not beat the human reference in terms of general translation quality; the human reference has been judged to be significantly better. Of course, we are missing here the split into adequacy
and fluency that the authors propose, but a comment from the authors on that part would be direly needed as it questions the repeatability of their results for newer or other test sets even in the news domain, not to mention other domains.
We have added comparison with WMT19 English→Czech news task into the discussion:

During reviews of this manuscript, the WMT19 competition took place. The testing dataset was different, and evaluation methodology was innovated compared to WMT18, which is

why the results are not directly comparable (e.g., the translation company was explicitly instructed to not add/remove information from the translated sentences, which was a major source of adequacy errors in this study (Fig. 4A)). Based on discussions with our team's members, the organizers of WMT19 implemented a context-aware evaluation (although a different one than we use in our study, see below). In this context-aware evaluation of English→Czech news task, CUNI-Transformer=CUBBITT was the winning MT system and reached overall quality score 95.3% of human translators (DA score 86.9 vs 91.2), which is similar to our study (94.8%, mean overall quality 7.4 vs 7.8, all evaluators together). Given that WMT19 did not separate overall quality into adequacy and fluency, it is not possible to validate the potential super-human adequacy on their dataset.

Lines 310-313 are such a strongly worded claim which is problematic the moment the authors step out of the news domain for which these systems were specifically built. I agree that human translators are not necessarily the upper bound for translation quality, but that has been shown only for one language pair, for one test set in one domain. Generalizing from that alone seems overconfident. The WMT2019 results across multiple languages, especially en-de and de-en, might give that more credence as a full picture, but for that they would have to be at least mentioned in this paper.
We have removed the last sentence of that paragraph. We have also added a paragraph on the generalisation to other languages into the discussion:

Our study was performed on English→Czech news articles and we have also validated the methodological improvements of CUBBITT using automatic metric on English↔French and English↔Polish news articles. The generality of CUBBITT's success with regards to other language pairs and domains remains to be evaluated. However, the recent results from WMT19 on English→German show that indeed also in other languages the human reference is not necessarily the upper bound of translation quality.

The other big question which I find has not been answered to my full satisfaction is how has the feat of superhuman quality actually been achieved? I would have liked to see a verification of the iterative block back-translation method for other languages than just translations between Czech and English, even if done using automatic metrics. It seems this is the only meaningful improvement presented in this paper over other transformer-based baselines, so it would be solely responsible for achieving superhuman translation quality (or for at least getting over that threshold)? Is that repeatable for other languages, for other domains? Is the Czech-English parallel training corpus somehow special? These things at least require more comments. The authors hint at the possibility that this is a compounding effect (314-322) of multiple factors but do so in the conclusions only.
We have now extended our manuscript with the following two major additions:

First, we have trained CUBBITT also for the English-French and English-Polish language pairs (both directions), evaluated it using automatic metric (BLEU), and observed similar

results as obtained on the English-Czech pair. In particular, the new language pairs also showed a synergy between block-BT and checkpoint averaging and the combined model in its peaks clearly outperformed mix-BT in all the four new language translation directions (Fig. S14).

Second, we have performed a sequence of analyses exploring how and why the synergy between block-backtranslation and checkpoint averaging works, suggesting it to be a general and language-independent principle. Please see the new section "Generality of block backtranslation and why does it improve translation?"). The results show that the alternation of authentic and synthetic data in block-BT leads to increased diversity of the translations (Fig. S16), which is leveraged by checkpoint averaging to generate novel translations, not used in the past by the model without averaging (Fig. 8A). We show that these novel translations largely contribute to the increased performance as measured by BLEU (Fig. 8B). Finally, we show how this synergy can work in concrete sentence examples. For instance, in Fig. 7A and S17, the translation produced by block-BT with checkpoint averaging contains two phrases which were correctly translated only by the models trained in the authentic blocks, while another phrase was correctly translated only by the models trained in the synthetic blocks. Only after the checkpoint averaging of both types of models, the system combined all three phrases correctly to form a good translation.

Smaller comments or questions (in no particular order):
- In the references I recommend replacing the Arxiv links with ACL Anthology links wherever possible. Many of the cited works have been published at conferences in parallel to Arxiv versions and these should be considered canonical.
We have updated the references according to your suggestion.

- What role might test set quality variability play considering the lack of superhuman results for English-Czech in WMT2019?
As we have described above (and in the new discussion), the comparison of the *overall quality* of CUBBITT vs human reference is not too different in our evaluation and in WMT19. It would be very interesting to see whether the superhuman *adequacy* of CUBBITT would be validated, however, unfortunately, it has not been evaluated in WMT19.

Nevertheless, it is likely that variability of the reference translation will influence any results of evaluation of MT vs human translation. We now discuss the importance of the reference variability and quality in the penultimate paragraph of the Discussion.

- How does current evaluation practice of WMT2019 differ from the one presented?
In WMT19, also based on discussions with our team's members, the organizers of WMT19 decided to make the evaluation context-aware (compared to the previous years). However, there were important differences in the evaluation setup. In WMT19, the

evaluators were first asked to score individual sentences, shown in their original sequential order as in the original document, one sentence per screen, and the evaluators could not return back. This was followed by a screen with the entire document, where the evaluators were asked to give one value for the document. Conversely, our evaluation was implemented in a spreadsheet, where the evaluators saw all sentences in the document and could therefore re-read it as many times as needed. We consider our approach to be fairer, as it allows correct evaluation of context-dependent issues that may not be obvious on the first sight: such as gender/named entity/ambiguous word translation (which can be resolved only with knowledge of a distant document context), or addition/omission of a fact in a sentence that was also mentioned a long time ago in the document (and is not a true error). Finally, our evaluators could see sentences ahead of the translated sentence, unlike the evaluators in WMT19, which is also important for correct evaluation of context-based errors.

The second difference between WMT19 and our study lies in the evaluation strategy. In WMT19, the evaluators were always asked to score only translation by one MT system (or human reference) at a time, giving it a "direct assessment score" between 0-100. Conversely, in our evaluation, translations by all the evaluated systems were shown side-by-side for every sentence. We believe that this allows more direct comparison of the different translations (although we understand that adopting our approach by WMT would be complicated due to the large number of evaluated systems in WMT).

Third, WMT19 evaluated only the overall quality of the translation. We also evaluated adequacy, fluency, and classification of the individual translation errors.

Fourth, WMT19 used a combination of crowd-sourcing and volunteers hired by the WMT19 participants (called "researchers"). We hired paid evaluators. Both WMT19 and our evaluation required the evaluators to be native Czech speakers fluent in English. WMT19 had no additional requirements (except for the quality control mentioned below) on the evaluators. Our evaluation involved in addition to non-professionals also six professional translators (with at least eight years of professional experience) and three translation theoreticians.

The exact strategy of quality control of the evaluators was also different to an extent, partially resulting from the differences explained above, but in both cases involved inclusion of a small number of translations of intentionally bad quality ("bad reference" in WMT19, "spam documents" in our study) and some form of inter-annotator agreement quantification.

Similarly as in our evaluation, the organizers of WMT19 have decided to include only original English sentences in the test set.

- Please provide a date for when your translations have been collected from Google Translate etc. They are likely to change over time and should be marked with something like a timestamp.

The Google Translate translations in our human evaluation of five MT systems (Fig. 4B) were identical to the onlineB translations in WMT18. The Google Translate translations in our Turing test evaluation were collected on 13th August 2018. We have now added this information into the Supplementary Materials.

- Lines 153-154 need to makes clear that this is the case for the language pairs investigated, otherwise it sounds hyperbolic, since you we don't know what was going on for the other language pairs.

We have clarified this in the updated manuscript:

In 2018, CUBBITT won English→Czech and Czech→English news translation task in WMT18 (17), surpassing not only its machine competitors, but it was also the only MT system which significantly outperformed the reference human translation by a professional agency <u>in WMT18 English→Czech news translation task (other language pairs were not evaluated in such a way to allow comparison with the human reference)</u> (Fig 3A).

- The paper is well written and presented although the limitations of the journal seem to have resulted in moving many important parts into the supplementary material. This seems to make the paper a bit incomplete without them: one such example is the so-called "translationese tuning" which is not clear without consulting the supplementary. This is a minor point.

We agree that the Supplementary Materials are an important part of the manuscript and that readers from the computer science field may not be used to this division. We have tried to put all the essential information into the main text of the manuscript, while keeping it concise and readable for the general readership, and thoroughly explain the more technical parts and other details in the Supplementary Materials. We consider the "translationese tuning" to be more on the technical side, not essential for the understanding of the results, and therefore explained it only in the Supplementary Materials (rather than trying to include a brief and incomplete description in the main text).

Moreover, we have now included more references to the Supplementary Materials, including the actual section numbers, which we hope will help the readers to find the methodological details and explanations as quickly as possible.

My final comment would be: this is a good paper, but as it stands now, it has been overtaken by the developments of 2019 and needs updating before publication. The results need to be put into a larger context, including specifically results from WMT2019.

As described above, we have updated our manuscript with discussion of the development of 2019, which happened while our manuscript was in review, and with substantial additional analyses that give insight into the generality of our findings and mechanisms of the method, based on the very useful comments suggested by both reviewers.

We would like to thank both the reviewers for their detailed feedback, which has greatly improved the clarity and depth of our study.

REVIEWERS' COMMENTS:

Reviewer #1 (Remarks to the Author):

The authors have extensively addressed my concerns.

I do think that the point where we can say with a degree of certainty that machine translations are better quality than professional translations, is an important milestone. My earlier concern about the quality of professional translations being the main issue here, not the quality of machine translation, is of course always an issue. But the authors have argued convincingly that affordable human translations will likely never be perfect and it is fine to compare MT to them.

The summary sentence is poorly written line 31: Open-source neural network CUBBITT shows how a professional agency translation can be outperformed in accuracy of news translation

> REVIEWERS' COMMENTS:
>
> Reviewer #1 (Remarks to the Author):
>
> The authors have extensively addressed my concerns.
>
> I do think that the point where we can say with a degree of certainty that
> machine translations are better quality than professional translations, is an
> important milestone. My earlier concern about the quality of professional
> translations being the main issue here, not the quality of machine translation,
> is of course always an issue. But the authors have argued convincingly that
> affordable human translations will likely never be perfect and it is fine to
> compare MT to them.
>
> The summary sentence is poorly written line 31: Open-source neural network
> CUBBITT shows how a professional agency translation can be outperformed in
> accuracy of news translation

We removed the sentence altogether, as its purpose is better served by the editor’s summary.
